# Co-targeting TGF-**β** and PD-L1 sensitizes triple-negative breast cancer to experimental immunogenic cisplatin-eribulin chemotherapy doublet

**DOI:** 10.1172/JCI184422

**Published:** 2025-07-01

**Authors:** Laura Kalfeist, Fanny Ledys, Stacy Petit, Cyriane Poirrier, Samia Kada Mohammed, Loïck Galland, Valentin Derangère, Alis Ilie, David Rageot, Romain Aucagne, Pierre-Simon Bellaye, Caroline Truntzer, Marion Thibaudin, Mickaël Rialland, François Ghiringhelli, Emeric Limagne, Sylvain Ladoire

**Affiliations:** 1 Université de Bourgogne Europe, Dijon, France.; 2 Cancer Biology Transfer Platform, Centre Georges-François Leclerc, INSERM UMS 58 BioSanD, Equipe Labellisée Ligue Contre le Cancer, Dijon, France.; 3 INSERM UMR 1231, Center for Translational and Molecular Medicine (CTM), Dijon, France.; 4 Genetic and Immunology Medical Institute, Dijon, France.; 5 Department of Medical Oncology, Centre Georges-François Leclerc, Dijon, France.; 6 Unit for Innovation in Genetics and Epigenetics in Oncology, Dijon University Hospital, Dijon, France.; 7 INSERM UMS 58 BioSanD, CRISPR Functional Genomics (CRIGEN) Facility, Dijon, France.; 8 Plateforme d’imagerie et de radiothérapie précliniques, INSERM UMS 58 BioSanD, Centre Georges-François Leclerc, Dijon, France.

**Keywords:** Immunology, Oncology, Breast cancer, Cancer immunotherapy

## Abstract

In preclinical mouse models of triple-negative breast cancer (TNBC), we show that a combination of chemotherapy with cisplatin (CDDP) and eribulin (Eri) was additive from an immunological point of view and was accompanied by the induction of an intratumoral immune and inflammatory response favored by the immunogenic cell death induced by CDDP, as well as by the vascular and tumor stromal remodeling induced by each chemotherapy. Unexpectedly, despite the favorable immune context created by this immunomodulatory chemotherapy combination, our models remained refractory to the addition of anti–PD-L1 immunotherapy. These surprising observations led us to discover that CDDP chemotherapy was simultaneously responsible for the production of TGF-β by several populations of cells present in tumors, which favored the emergence of different subpopulations of immune cells and cancer-associated fibroblasts characterized by immunosuppressive properties. Accordingly, co-treatment with anti–TGF-β restored the immunological synergy between this immunogenic doublet of chemotherapy and anti–PD-L1 in a CD8-dependent manner. Translational studies revealed the unfavorable prognostic effect of the TGF-β pathway on the immune response in human TNBC, as well as the ability of CDDP to induce this cytokine also in human TNBC cell lines, thus highlighting the clinical relevance of targeting TGF-β in the context of human TNBC treated with chemoimmunotherapy.

## Abstract 2

This article proposes a biological rationale highlighting the value of using a combination of immunotherapy targeting PD-L1 and TGFb with a new cisplatin and eribulin-based chemotherapy doublet in breast cancer.

## Graphical abstract

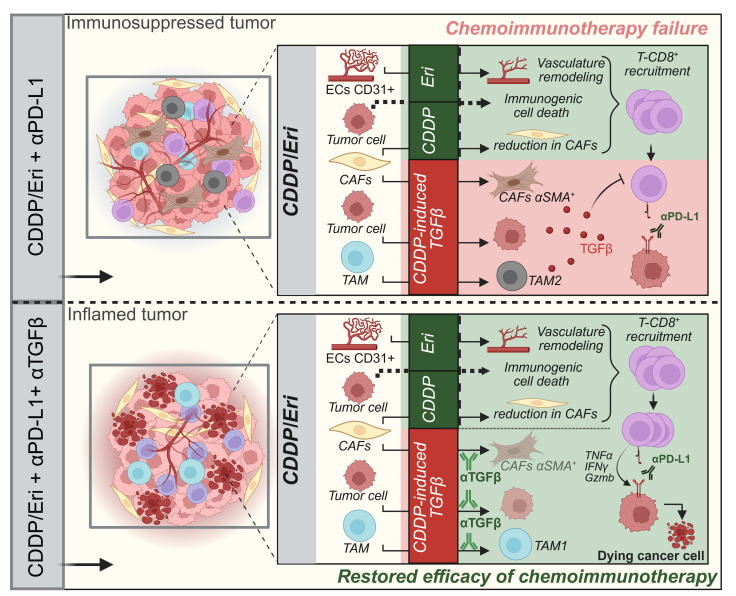

## Introduction

From an immunological viewpoint, triple-negative breast cancer (TNBC) is currently considered the most immunogenic breast cancer (BC) subtype due to the high infiltration by tumor-infiltrating lymphocytes (TILs), more frequent tumor expression of programmed death–ligand 1 (PD-L1), and greater tumor mutational burden (TMB) compared with other forms of BC (1).

However, in the metastatic setting, only a minority of patients actually show a benefit from the combination of immunotherapy and chemotherapy (2). Recent translational research studies in the setting of TNBC have shown that several patient groups received less benefit from anti–PD-(L)1 therapy, namely patients with “cold” tumors (i.e., tumors not infiltrated by immune cells and showing no signs of an immune response), those with an immune-excluded phenotype (i.e., the immune reaction remains outside of the tumor core), or patients with tumors with an immune-suppressed signature (3, 4). There is thus a compelling need to advance our understanding of several unresolved questions, namely: (a) why some patients do not respond to the combination of chemotherapy and anti–PD-(L)1 and (b) which are the optimal chemotherapy agents to combine with anti–PD-(L)1 immunotherapy, with a view to enhancing the response to and efficacy of this combination.

Several preclinical and clinical studies have shown that certain chemotherapy agents may exert their antitumor effects by enhancing the antitumor immune response (5). These immunogenic effects of chemotherapy can be exerted by inducing immunogenic cell death (ICD) (thus enabling the release of damage-associated molecular patterns [DAMPs] in the tumor microenvironment [TME] or the release of chemokines to recruit immune cells). The antitumor efficacy of chemotherapy may also be mediated by countering immune-suppressive mechanisms, such as selective elimination of certain immunosuppressive populations, e.g., Tregs or myeloid-derived suppressor cells (MDSCs) (5). Among the chemotherapy agents, anthracyclines (doxorubicin [DXR], epirubicin) and taxanes (paclitaxel, docetaxel) are the most widely used to treat BC, particularly TNBC, at all stages of the disease. The immunological effects of these chemotherapy agents have previously been described, in both preclinical models and in patients with BC (especially regarding the induction of ICD by anthracyclines) (5). Studies investigating taxanes have reported less marked effects on the immune response, sometimes with divergent results (6). Nevertheless, despite the lack of a formal demonstration of immunological synergy with anti–PD-(L)1, taxanes remain the family of chemotherapy agents most widely used as a first-line therapy in metastatic TNBC (mTNBC) in association with immunotherapy, be it in clinical trials or in routine practice.

Other chemotherapy agents used for the treatment of mTNBC (usually as a second-line agent) include platinum salts (cisplatin [CDDP]) and eribulin (Eri). Platinum salts are frequently prescribed in mTNBC due to the frequency of defects in homologous recombination in this BC subtype (7). Eri mesylate is a microtubule-targeting agent that has become a key treatment in metastatic BC, especially for patients with mTNBC, who have been shown to have a survival benefit with this chemotherapy (8). The immunological effects of these 2 chemotherapies have not been widely investigated. However, in translational analyses of the TONIC study (9), CDDP seemed to be capable of inducing an intratumoral immune response conducive to the action of an anti–PD-(L)1 in some patients with mTNBC. Furthermore, in estrogen receptor–positive BC, although there does not appear to be synergy between Eri and pembrolizumab (an anti–PD-1 agent), the existence of an inflamed tumor signature is associated with a clinical benefit of Eri monotherapy, suggesting a possible interaction between this molecule and the antitumor immune response (10).

Here, using several preclinical models of TNBC unfavorable to the efficacy of immunotherapy (i.e., cold and immune-excluded tumor models), we investigated the potential immunogenic effects of CDDP and Eri, alone or in combination (CDDP-Eri).

We show that the combination of CDDP-Eri was immunologically additive in vivo in terms of recruitment, activation, and maintenance of an intratumoral immune response. However, this immunological synergy was not accompanied by a greater antitumor efficacy, and did not provide therapeutic synergy in association with an anti–PD-L1 antibody. Importantly, we show that immunological “heating up” of the tumor induced by the combination of CDDP and Eri, was accompanied by early production of TFG-β in the tumor (mainly driven by CDDP treatment), whose multiple immunosuppressive effects counterbalanced the action of the immune response. Blockade of TGF-β in association with the 2 chemotherapeutic agents plus an anti–PD-L1 made it possible to enhance the efficacy of the immune response and tumor control.

These results provide a rationale to develop co-targeting of PD-L1 and TGF-β, in association with this efficient cytotoxic-immunogenic chemotherapy doublet, in the context of refractory/relapsing mTNBC with an unfavorable immunophenotype.

## Results

### The CDDP-Eri chemotherapy combination shows no antitumor synergy, despite the additive immunological effect in TNBC models.

**Figure 1:**
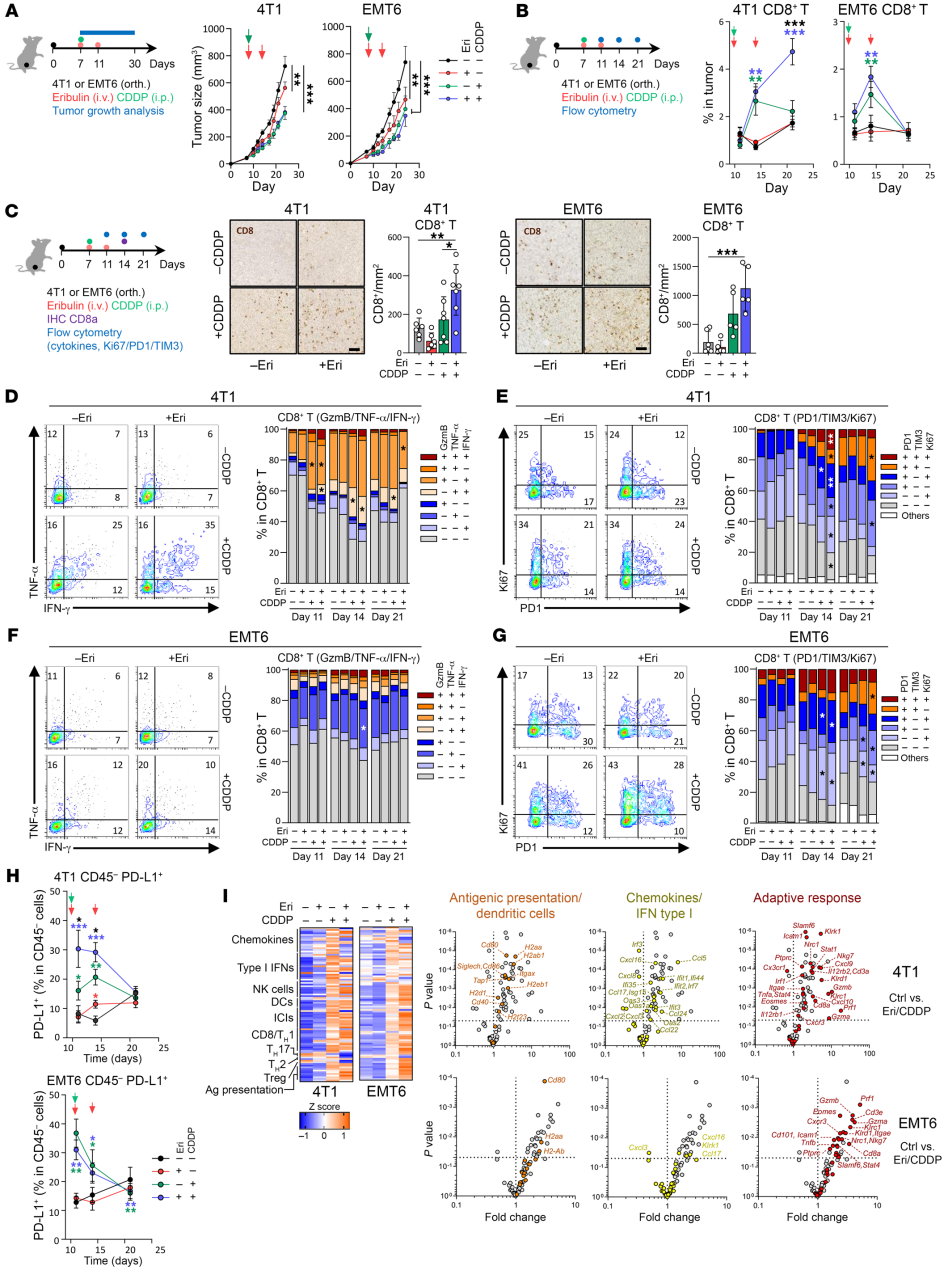
The CDDP-Eri doublet increases tumor CTL infiltration and functionality. (**A**) 4T1 and EMT6 tumor–bearing mice were treated with CDDP, Eri, or CDDP-Eri, and tumor volume was monitored for 30 days (*n* ≥10 for 4T1; *n* ≥5 for EMT6). (**B**) CTL proportions were assessed by flow cytometry in tumors collected at days 4, 8, and 14 after treatment (*n* ≥5/group). (**C**) At day 8, CTLs were visualized by IHC and quantified using QPath (scale bars: 200 μm; *n* ≥6 for 4T1; *n* ≥5 for EMT6). Images are shown again in Supplemental Figure 2C. (**D** and **F**) CTL functionality was evaluated by measuring GzmB, TNF-α, and IFN-γ expression in 4T1 (**D**) and EMT6 (**F**) tumors. Representative dot plots and mean values are shown. (**E** and **G**) Activation/proliferation markers (PD-1, TIM-3, and Ki67) were analyzed in 4T1 (**E**) and EMT6 (**G**) tumors using flow cytometry, with representative dot plots at day 8 (left) and mean values (right). (**H**) PD-L1 expression on CD45^−^ tumor cells was quantified by flow cytometry (*n* ≥4/group). (**I**) Immune-related gene expression was assessed by NanoString for total tumor mRNA. Heatmap shows normalized *z* scores, and volcano plots indicate statistical significance (*n* = 6/group). Box plots show the mean ± SEM. *P < 0.05, **P < 0.01, and ***P < 0.001, by 2-way ANOVA. orth., orthotopic.

First, we evaluated the antitumor and immunological effects of CDDP and Eri, alone or in combination, in 4T1 and EMT6 orthotopic murine models of TNBC presenting distinct tumor immune microenvironment profiles, both unfavorable to the efficacy of PD-L1 blockade compared with the “hot” MC38 model. 4T1 is an immunologically “cold” TNBC model, characterized by low tumoral infiltrates of CD8^+^ T lymphocytes (CTLs) and low intratumoral PD-L1 expression (Supplemental Figure 1, A–D; supplemental material available online with this article; https://doi.org/10.1172/JCI184422DS1). EMT6 is an “immuno-excluded” TNBC model, characterized by equivalent PD-L1 expression and greater CTL infiltration, but limited to the tumor periphery (Supplemental Figure 1, A–D). In vivo, in our 2 models, monotherapy with Eri or CDDP had acceptable and transient toxicity in animals (Supplemental Figure 2A) and a significant but modest antitumor effect by slowing tumor growth (Figure 1A). Combining the 2 molecules was not more toxic than CDDP monotherapy (Supplemental Figure 2A) and did not confer antitumor therapeutic synergy compared with CDDP alone (Figure 1A).

On the other hand, from an immunological perspective, in our 2 models, treatment with CDDP alone, but especially the CDDP-Eri combination, was accompanied by an increase in tumor infiltration with CTLs (Figure 1, B and C). This intratumoral influx of CTLs occurred early after treatment (as early as day 8 and peaking at day 21 in 4T1 and day 14 in EMT6 tumors), and was significantly greater after treatment with the CDDP-Eri combination compared with each monotherapy (Figure 1, B and C). Study of the phenotype of these tumor-affluent CTLs in both models showed that the percentage of polyfunctional T cells (expression or coexpression of IFN-γ, granzyme B [GzmB], TNF-α) (Figure 1, D and F), proliferating T cells (Ki67^+^) and expression of markers of lymphocyte activation/exhaustion (PD-1/TIM3) (Figure 1, E and G) were also greater in tumors treated with the CDDP-Eri combination, particularly at early time points (day 14). At the same time, the proportion of PD-L1^+^ tumor cells was also greater after CDDP-Eri treatment (Figure 1H). More broadly, study of the tumor bulk transcriptome (in particular the expression of genes involved in the immune response) of 4T1 and EMT6 tumors after different treatments showed little change for tumors treated with Eri alone, but did show the induction of numerous immune genes after treatment with CDDP, especially after CDDP-Eri treatment (Figure 1I). Treatment with CDDP-Eri was accompanied by intratumoral induction of genes involved in (a) immune cell recruitment (*Ccl5*, *Ccl22*, *Ccl17*, *Cxcl9/10*); (b) type I IFN response (*Ifi35*, *Ifit2*); (c) NK cell response (*Klrk1*, *Klrd1*, *Klrc1*); (d) co-stimulation signals (*Cd40*, *Cd80/86*); (e) immune checkpoint inhibitors (ICIs) (*Pdcd1*, *Ctla4*); (f) cytotoxicity (*Prf1*, *Gzma/b*, *Stat1*, *Irf1*); (g) T cell response (*Cd3e*, *Cd8a*); and (h) antigen presentation (*Tap2*, *H2ab1*) (Figure 1I). Comparison of the different treatments with regard to their effects on the tumor transcriptome showed that this induction of immune response gene expression was driven mainly by CDDP (particularly in the 4T1 cold model), and that the combination of CDDP-Eri enhanced this effect, particularly with regard to the expression of genes involved in antigen presentation, the type I IFN response, or the recruitment of immune cells involved in the adaptive response (Supplemental Figure 2B). In the 2 models, this recruitment of CTLs with the CDDP-Eri combination was observed not only at the tumor invasion front, but also within the tumor, in contact with tumor cells (Supplemental Figure 2C).

Taken together, these results show that, in our mouse models of TNBC, the CDDP-Eri combination did not result in antitumor synergy but did appear to be immunologically additive, inducing a global inflammatory and immune tumor response, accompanied by intratumoral PD-L1 expression, as well as intratumoral recruitment of activated, cytotoxic, proliferative, and polyfunctional CTLs.

### CDDP and Eri do not have the same immunogenic (ICD) and antigenic properties and have different effects on tumor stromal remodeling.

In order to better understand the immunological effects of CDDP and Eri and to understand their immunological additivity observed in vivo, we first sought to assess the ability of these chemotherapies, alone or in combination, to induce immunogenic death of tumor cells (ICD). The molecular steps involved in ICD are now well described and consist of changes in plasma membrane composition and the release of soluble mediators by tumor cells, which are then able to stimulate an immune response against the treated tumor (11). ICD has been well described with certain chemotherapies such as DXR. In contrast, the ability of CDDP and Eri to induce ICD and antigenicity in TNBC tumor cells has not yet, to our knowledge, been described.

**Figure 2:**
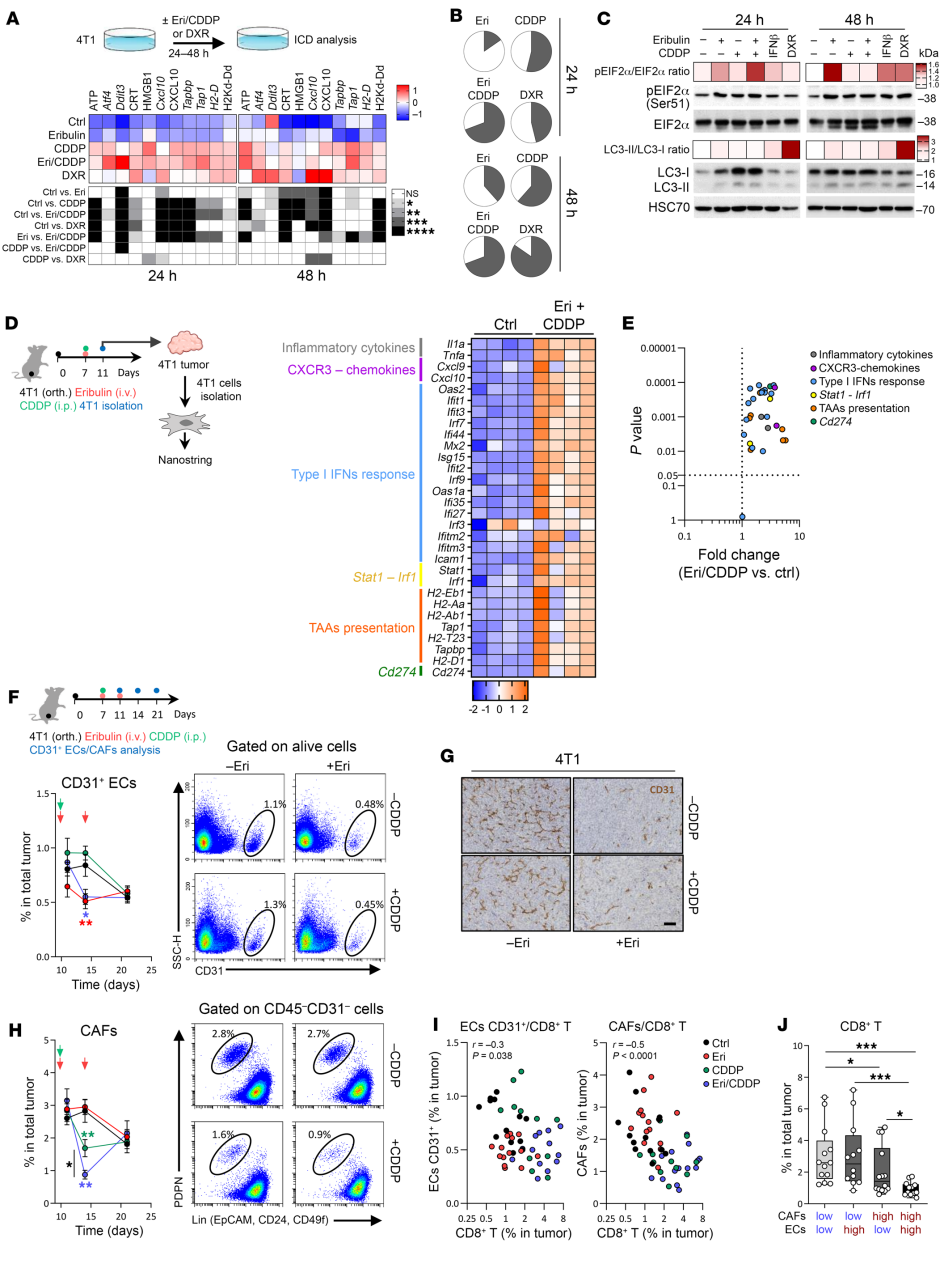
The CDDP-Eri doublet induces ICD markers. (**A**) 4T1 cells were treated with Eri (50 nM), CDDP (4 μM), DXR (500 nM), or CDDP-Eri. Drug concentrations correspond to the IC_50_ at 48 hours. ICD markers were analyzed at 24 hours and 48 hours. Heatmap shows normalized marker expression and statistical significance. (**B**) Pie charts indicate the proportion of ICD marker positivity across independent experiments (1-way ANOVA). (**C**) EIF2α phosphorylation (Ser51) and LC3I/II levels were assessed by Western blotting. Heatmaps represent the densitometric phosphorylated/total ratio (1 representative experiment of 2). (**D** and **E**) Experimental design. 4T1 tumor–bearing mice received CDDP, Eri, or both. Four days later, tumor cells were isolated for NanoString analysis. Heatmap in **D** shows normalized gene expression; volcano plot in **E** indicates statistical significance (*n* = 4/group); 1-way ANOVA. (**F**) CD31^+^ endothelial cell proportions were measured by flow cytometry on post-treatment days 4, 8, and 14. Dot plots show CD31^+^ populations at day 8 (*n* ≥ 6/group). (**G**) CD31^+^ cells were quantified by IHC using QPath (scale bar: 200 μm). (**H**) CAF proportions were assessed according to PDPN expression using flow cytometry on day 8. Dot plots represent PDPN^+^ cells (*n* ≥ 6/group). (**I**) Correlations between CD31^+^, CAF, and CTL proportions were analyzed (*n* ≥ 11). (**J**) CTL proportions were evaluated on the basis of CAF and CD31^+^ levels (> median = high, < median = low). Box plots show the mean ± SEM. *P < 0.05, **P < 0.01, ***P < 0.001, and ****P < 0.0001, by 2-way ANOVA. Ctrl, control.

We then standardized in vitro experiments in which each chemotherapy (CDDP, Eri, CDDP-Eri, or DXR) induces approximately 50% cell death after 48 hours of treatment (Supplemental Figure 3, A–F) and determined their immunogenic properties (4T1 model: Figure 2, A–C, EMT6 model: Supplemental Figure 3, G and H). We studied the induction by chemotherapies of 11 biological elements linked to ICD (reticulum stress: calreticulin [CRT], *Ddit3*, *Atf4*; purinergic signaling: ATP; TLR4 signaling: HMGB1; type I IFN and chemokines: *Cxcl10*; antigenic presentation and antigenicity: H2-D, H2Kd/Dd, *Tap1*, *Tapbp*). We also studied the induction of EIF2α phosphorylation and the LC3-I/LC3-II ratio. DXR was used as a positive control. We observed in our 2 models that Eri alone had little or no ability to induce immunogenic signals compared with controls (Figure 2, A–C, and Supplemental Figure 3, G and H). On the contrary, CDDP induced numerous immunogenic death signals at 24 hours, and some of these signals persisted at 48 hours. In our 2 models, CDDP induced as many or more early ICD signals (24 hours) as those induced by DXR treatment. These effects of CDDP were still observed at 48 hours, as was the ICD induced by DXR. The CDDP-Eri combination was able to induce a greater number of early ICD signals than with DXR, again with an effect maintained at 48 hours. In contrast, the CDDP-Eri combination did not appear to induce more ICD signals than CDDP alone (except marginally on a few elements in the EMT6 model). In line with these results, the transcriptome of in vivo–treated 4T1 tumor cells considerably changed after treatment with the CDDP-Eri combination, showing the induction of the expression of numerous proinflammatory or chemoattractant cytokine genes, genes involved in the type I IFN response or CTL/Th1 polarization, genes involved in antigenic presentation, as well as genes involved in PD-L1 (*Cd274*) induction (Figure 2, D and E). We then sought to examine the effects of CDDP, Eri, and their combination on other TME cellular subsets, in particular those making up the vascular and connective compartments, by studying cancer-associated fibroblasts (CAFs) (Supplemental Figure 3I). We thus demonstrated that Eri (alone or in combination) had an antivascular effect in our models by depleting CD31^+^ cells (Figure 2F), which was accompanied morphologically by a reduction in tumor vessel density (Figure 2G). On the other hand, CDDP (alone or in combination) had no effect on tumor vasculature. In the case of CAFs, however, we observed that CDDP had a depleting effect on the pool of these cells and that the combination with Eri further increased this effect (Figure 2H and Supplemental Figure 2I).

These treatment-induced changes in TME cell populations were strongly correlated with CTL infiltration: there was a significant association between high CTL infiltration and (a) a low number of CD31^+^ cells induced by Eri (alone or in combination with CDDP) (Figure 2I) and (b) a low number of CAFs induced by CDDP (Figure 2I). These results therefore suggest a favorable role for the effects of the CDDP-Eri combination on the TME in the induction of an intratumoral immune response. Thus, the tumors most infiltrated with CTLs after treatment wee also those in which a low percentage of CD31^+^ cells was associated with a low percentage of CAFs (Figure 2J). Importantly, when the 2 chemotherapies were combined, the antiangiogenic effects of Eri were not responsible for a better penetration of CDDP into tumors nor therefore, for a higher concentration of intratumoral immunogenic platinum compounds (Supplemental Figure 2J).

Finally, unlike Eri, which did not induce an ICD stigma, in our TNBC mouse models, CDDP proved to be a chemotherapeutic agent capable of inducing as many ICD signals as those induced by DXR. The CDDP-Eri combination did not appear to induce more ICD signals than CDDP alone. Therefore, ICD alone did not explain the immunological synergy of the CDDP-Eri combination previously observed in vivo. In contrast, CDDP and Eri had separate effects on CAFs and the tumor vasculature, respectively, which, when the 2 molecules were combined, seemed to correlate with the intensity of intratumoral immune recruitment.

### The antitumor effect of CDDP-Eri combination does not depend on the CTL response and does not synergize with anti–PD-L1 immunotherapy.

We then investigated whether the inflammatory response and recruitment of immune cells induced by the CDDP-Eri combination in our models contributed to the antitumor efficacy of the combination of this association. To this end, we evaluated the effects on tumor growth in the 4T1 model of treatment with CDDP, Eri, or the combination of both in immunocompetent mice, and then nude (nu/nu) mice, and in mice treated with an anti-CD8–depleting antibody.

**Figure 3:**
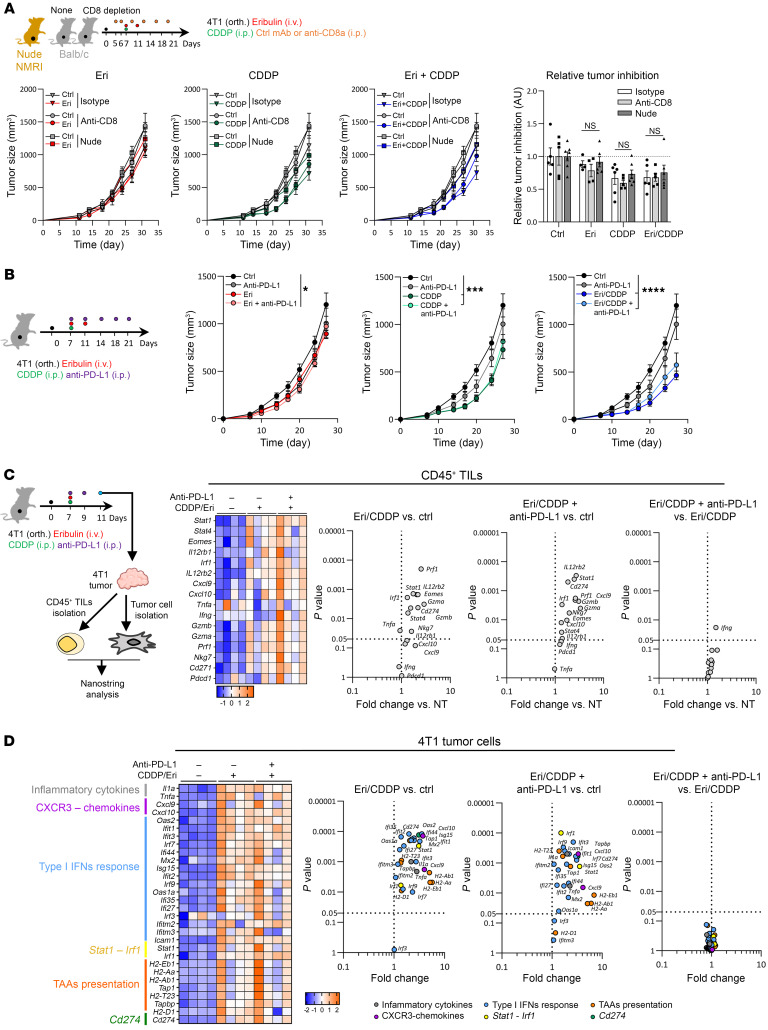
Combination therapy is CTL independent and resistant to anti–PD-L1 immunotherapy. (**A**) 4T1 tumor–bearing (orth) BALB/c immunocompetent or nude mice received CDDP, Eri, or CDDP-Eri, with or without anti-CD8a or isotype control antibodies. The anti-CD8a antibody was injected twice before and 4 times after chemotherapy. Tumor volume was monitored for at least 3 weeks after treatment (*n* = at least 4 mice/group). (**B**) 4T1 tumor–bearing mice received CDDP, Eri, or both, with or without anti–PD-L1 or isotype control antibodies. Tumor volume was monitored for at least 3 weeks after treatment (*n* = at least 6 mice per group). Box plots show the mean ± SEM. *P < 0.05, ***P < 0.001, and ****P < 0.0001, by 2-way ANOVA. (**C** and **D**) 4T1 tumor–bearing mice received CDDP, Eri, or both. Four days later, the 4T1 tumor was recovered, and tumor cells and CD45^+^ cells were isolated by magnetic beads. Total cell mRNA was extracted, and immune-related gene expression was analyzed by NanoString for CD45^+^ TILS (**C**) and tumor cells (**D**). The heatmap corresponds to normalized marker expression, and the volcano plot indicates the *P* value from the statistical analysis (*n* = 4 mice/group). aCD8, anti-CD8 antibody; aPD-L1, anti–PD-L1 antibody.

Compared with the immunocompetent animals, in the 4T1 model, we observed a preserved antitumor effect of the different chemotherapy regimens (Eri, CDDP, or the CDDP-Eri combination), despite the absence of T cells or the depletion of CTLs (Figure 3A), suggesting that the immunological synergy induced by the CDDP-Eri combination did not contribute to tumor growth control in the 4T1 model. Similarly, in the 4T1 model (totally unresponsive to anti–PD-L1 monotherapy), the combination of anti–PD-L1 immunotherapy with chemotherapy showed no synergy compared with chemotherapy alone, whether with Eri, CDDP, or the CDDP-Eri combination (Figure 3B), despite the fact that the CDDP-Eri combination was accompanied by polyfunctional PD-1^+^–activated CTL recruitment and induction of PD-L1 expression. A separate analysis of CD45^+^ TILs (Figure 3C) and of 4T1 tumor cells (Figure 3D) after treatment confirmed that the induction of the expression of genes associated with antitumor immune responses was uniquely linked to an effect of the CDDP-Eri combination, and that the addition of anti–PD-L1 in this context changed nothing in these 2 cell populations.

Thus, despite the favorable immunological effects observed in the immunologically “cold” 4T1 model, the inflammatory and immune responses induced by the CDDP-Eri combination did not appear to contribute to the drugs’ antitumor effect. Paradoxically, this apparent recruitment and activation of immune effectors did not allow therapeutic synergy between the CDDP-Eri combination and anti–PD-L1 immunotherapy. These intriguing observations led us to investigate possible associated immunosuppressive mechanisms in this therapeutic context.

### The CDDP-Eri combination is also associated with a complex qualitative and quantitative remodeling of the TME under the effect of CDDP-induced TGF-β.

**Figure 4:**
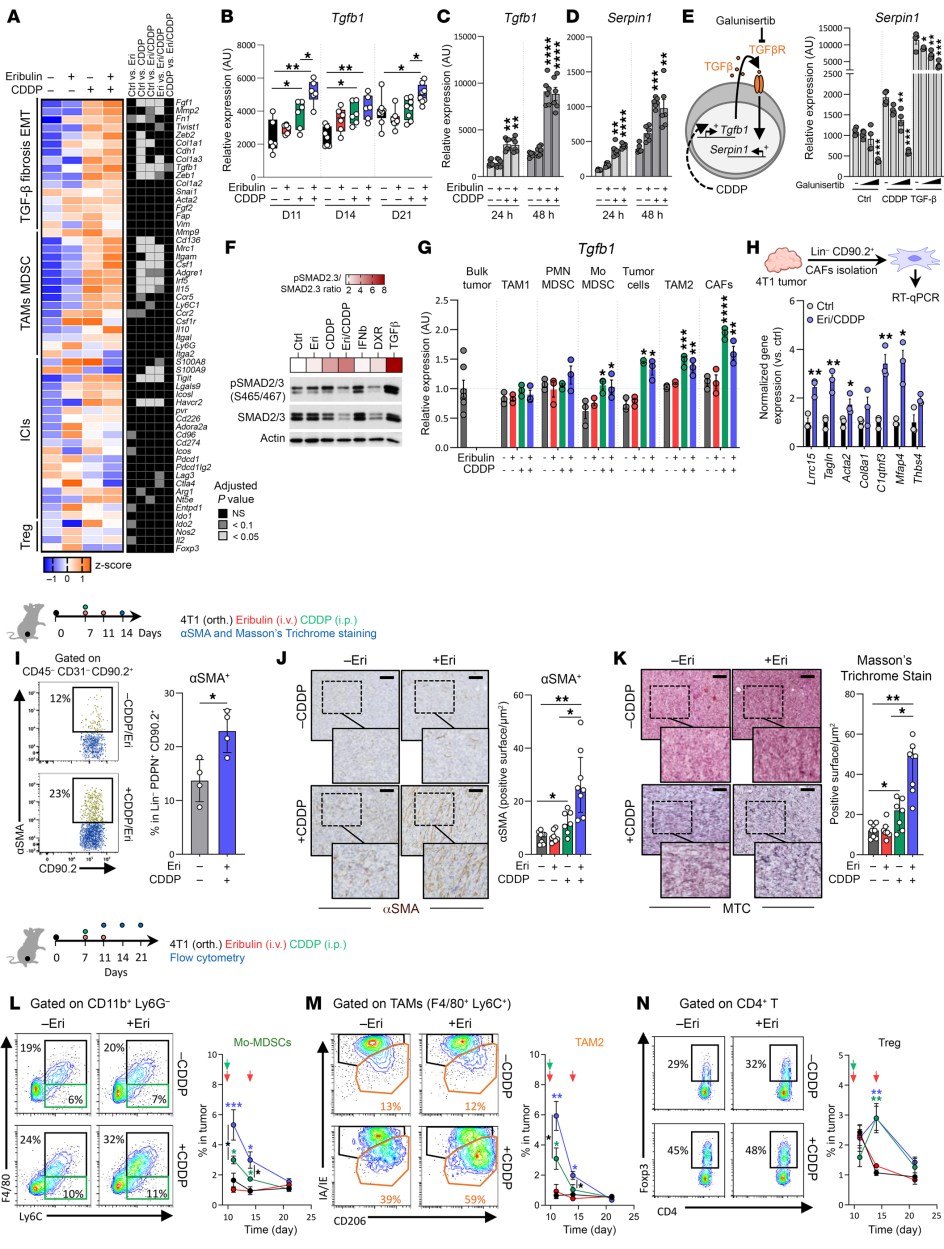
The CDDP-Eri doublet induces TGF-β–mediated immunoresistance. (**A**) 4T1 tumor–bearing mice were treated with CDDP, Eri, or both. Tumors were collected at days 4, 8, and 14. At day 8, total tumor mRNA was analyzed for immunosuppressive gene expression by NanoString. The heatmap on the left shows normalized expression, and the heatmap on the right shows statistical significance (*n* = 4/group). *P* values were determined by 2-way ANOVA. (**B**) *Tgfb1* expression was measured by qPCR at days (D) 4, 8, and 14 (*n* ≥6/group). (**C** and **D**) 4T1 cells were treated with Eri (50 nM), CDDP (4 μM), CDDP-Eri, or left untreated. *Tgfb1* (**C**) and *Serpin1* (**D**) expression was analyzed by qPCR at 24 hours and 48 hours (2 experiments, *n* = 3/experiment). (**E**) 4T1 cells were pretreated with galunisertib (10, 100, and 1,000 nM) for 2 hours, and then treated with CDDP (4 μM), TGF-β (2 ng/mL), or left untreated for 48 hours. *Serpin1* expression was analyzed by qPCR (2 experiments, *n* = 2/experiment). (**F**) Under the same conditions as in **C**, Smad2/3 phosphorylation [pSmad2 (Ser465/467), pSmad3 (Ser423/425)] was analyzed by Western blotting. Heatmap represents the phosphorylated/total ratio (results are from 1 of 2 experiments). (**G** and **H**) 4T1 tumor–bearing mice were treated as in **A**. At day 4, immunosuppressive cells were sorted, and *Tgfb1* expression was measured by qPCR (**G**). MyCAF-related genes were analyzed in CAFs (**H**) (*n* ≥3/group). (**I**) At day 8, αSMA^+^ CAF proportions were measured by flow cytometry. (**J** and **K**) αSMA^+^ cells were quantified by IHC (**J**), and collagen deposition was analyzed by Masson’s trichrome (MTC) staining (**K**) (*n* ≥7/group). Images are shown again in Supplemental Figure 4I. (**L**–**N**) Mo-MDSC (**L**), TAM2 (**M**), and Treg (**N**) proportions were assessed by flow cytometry (*n* = 5/group). Box plots represent the mean ± SEM. *P < 0.05, **P < 0.01, ***P < 0.001, and ****P < 0.0001, by 1-way ANOVA (**C**–**E**) and 2-way ANOVA (**B**, **G**, **H**, and **L**–**N**).

By studying changes in the tumor transcriptome (in particular the expression of genes involved in the main immunosubversion and immunosuppression pathways) after treatment with the different chemotherapies, we showed in the 4T1 model that the apparently favorable immunological effects initially observed were also accompanied, during treatment with the CDDP-Eri combination, by strong induction of genes involved in (a) the TGF-β pathway (*Tgfb1*); (b) fibrosis (*Col1a*, *Col3a*, *Fn1*, *Fgf1*); epithelial-mesenchymal transition (EMT) (*Zeb1*); (c) myeloid cell–mediated immunosuppression (tumor-associated macrophages [TAMs], MDSCs; *Cd163*, *Mcr1*, *Itgam*, *Csf1*, *Ly6c1*) (Figure 4A). These immunosuppression/immunosubversion pathway appeared to be much more influenced by CDDP-Eri treatment than by the expression of other immune response inhibitory checkpoints or genes involved in Treg biology (Figure 4A).

As the common factor in these pathways upregulated by CDDP-Eri treatment appeared to be TGF-β, we then confirmed in vivo *Tgfb1* gene induction in 4T1 tumors in CDDP-treated animals (Figure 4B). This phenomenon was also observed in vivo in the EMT6 model (Supplemental Figure 4A). We then investigated the sources of TGF-β produced in the treated tumors. In vitro, we showed induction of the *Tgfb1* gene in 4T1 (Figure 4C) and EMT6 (Supplemental Figure 4B) tumors 24 hours and 48 hours after CDDP and CDDP-Eri treatments. This TGF-β produced by tumor cells appeared to be biologically active, acting in an autocrine loop on tumor cells, since we observed induction of a TGF-β target gene (*Serpin1*) 24 hours and 48 hours after CDDP treatment in 4T1 (Figure 4D) and EMT6 (Supplemental Figure 4, B and C) tumors. To validate this observation, we tested the expression of *Serpin1* in CDDP-treated cells with or without galunisertib (a TGF-β receptor I [TGF-βRI] inhibitor). We observed that *Serpin1* expression was limited in a dose-dependent manner by galunisertib (Figure 4E and Supplemental Figure 4D). In addition, we also analyzed basal and chemotherapy-induced gene expression of *Tgfb2* and *Tgfb3*. We observed that *Tgfb2* was not expressed in 4T1 tumors and was not induced by chemotherapy. Finally, we found that *Tgfb3* was expressed to a lesser extent than *Tgfb1* and was not induced by treatment either (Supplemental Figure 4E). On the other hand, we observed that SMAD2/3 phosphorylation was increased in 4T1 tumor cells 48 hours after treatment with CDDP or CDDP-Eri (Figure 4F). The TGF-β at the origin of this autocrine loop appeared to be produced in active form by tumor cells, since its presence in the conditioned medium of CDDP-treated 4T1 cells previously induced luciferase activity in MLEC reporter cells (Supplemental Figure 4G). Interestingly, this induction of *Tgfb1* in 4T1 cells by CDDP appeared to be dose dependent and was also observed with carboplatin (another platinum salt commonly used for the treatment of TNBC) (Supplemental Figure 4H). Furthermore, after sorting different cell populations from the TME of 4T1 tumors, we also found that, apart from the tumor cells themselves, *Tgfb1* expression was also induced in monocytic MDSC (Mo-MDSC), TAM2, and CAF populations following treatment with CDDP and CDDP-Eri (Figure 4G). After treatment with CDDP or CDDP-Eri, CAFs isolated from 4T1 tumors had a more pronounced transcriptomic profile of LRRC15^+^ CAFs, a myofibroblast subset of CAFs (myCAFs), a population with immunosuppressive properties (12) recently described in abundance in BCs (13, 14) and belonging to TGF-β–induced CAF clusters (C0–C5 clusters) (15) (Figure 4H). Thus, we showed an increase in the percentage of myCAFs by analyzing α–smooth muscle actin (αSMA) expression in the total CD45^–^CD31^–^CD90.2^+^ CAF population in 4T1 tumors treated with CDDP-Eri (Figure 4I). This was reflected histologically by a higher density of αSMA^+^ spindle cells in 4T1 tumors after treatment with CDDP, and even more so after treatment with the CDDP-Eri combination (Figure 4J). Histologically, this was associated with a higher percentage of surface area of fibrous connective tissue in the tumors (Figure 4K), but without a CTL immuno-exclusion phenomenon (Supplemental Figure 4I). The biological phenomena observed in CAFs and associated with CDDP treatment in our models therefore appear complex, with, on the one hand, a depleting effect on the overall CAF population, but also the emergence of a TGF-β–responsive and immunosuppressive subpopulation.

Concerning the other cellular sources of chemotherapy-induced TGF-β that we identified in our models, we found that the percentage of Mo-MDSCs and TAM2s increased significantly and early (day 10) in CDDP-treated tumors, and again to a greater extent after treatment with the CDDP-Eri combination (Figure 4, L and M, and Supplemental Figure 4J). Among the other immunosuppressive cell populations analyzed, only Tregs increased in CDDP-treated tumors (Figure 4N and Supplemental Figure 4I), in agreement with the role of TGF-β in the proliferation of this cell population (16).

Taken together, these results show that treatment with the immunogenic CDDP-Eri combination was not only accompanied by significant recruitment of immune cells, but also by profound qualitative and quantitative changes in the TME, driven by substantial TGF-β production, which may explain certain immune-escape mechanisms, such as the accumulation of populations of immunosuppressive myeloid and lymphoid cells, as well as a subpopulation of immunosuppressive CAFs.

### Targeting TGF-β enhances the antitumor effect of the CDDP-Eri plus PD-L1 blockade combination and makes it CTL dependent.

To confirm the deleterious effect of TGF-β in our therapeutic models, we treated tumor-bearing mice (4T1, EMT6, as well as 2 other BC models: a luminal tumor model [TS/A], and a spontaneous model [mouse mammary tumor virus [MMTV] and polyomavirus middle T antigen [PYMT]) with an anti–TGF-β antibody. The aim was to test whether cotreatment with an anti–TGF-β antibody could make the immunogenic chemotherapy combination CDDP-Eri more effective when combined with anti–PD-L1 immunotherapy.

**Figure 5:**
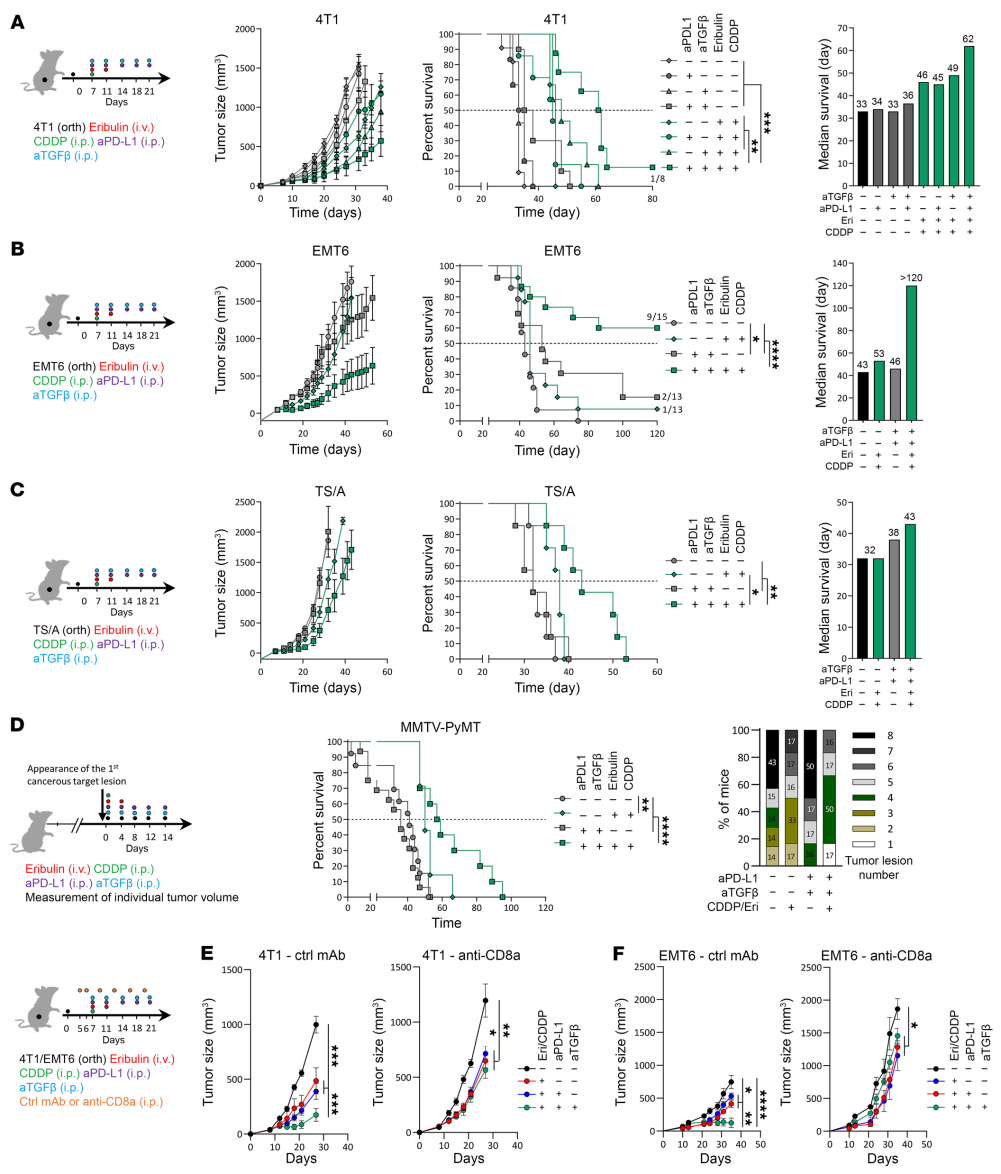
TGF–β blockade restores chemoimmunotherapy efficacy. (**A**–**C**) 4T1 (**A**), EMT6 (**B**), and TS/A (**C**) tumor–bearing mice were treated with the CDDP-Eri doublet, anti–PD-L1 mAb, and anti–TGF-β mAb, or the combination of the 4 molecules, or left untreated. Tumor volume was monitored (schema). Data represent the mean ± SEM. Mouse survival was evaluated (*n* = at least 5 mice/group). Data represent the median; log-rank test. (**D**) MMTV-PyMT mice were treated with CDDP-Eri doublet, anti–PD-L1, and anti–TGF-β mAbs, or the combination of the 4 molecules, or left untreated at the appearance of the first tumor target lesion. Mouse survival was evaluated for 120 days (control: *n* = 13, CDDP-Eri: *n* = 7, anti–PD-L1/anti–TGF-β: *n* = 16, quadritherapy: *n* = 10). Data represent the median; log-rank test. The number of tumors was assessed for each mouse at day 29 after the different chemotherapy treatments. (**E** and **F**) 4T1 (**E**) and EMT6 (**F**) tumor–bearing mice were treated with CDDP-Eri and CDDP-Eri plus anti–PD-L1 with or without anti–TGF-β mAbs with or without CTL depletion. Tumor volume was monitored for at least 4 weeks after treatment (*n* = at least 3 mice/group). Box plots show the mean ± SEM. *P < 0.05, **P < 0.01, ***P < 0.001, and ****P < 0.0001, by 2-way ANOVA.

In the 4T1 model, these therapeutic combinations did not appear to show any additional toxicity compared with the other therapeutic combinations used previously (Supplemental Figure 5A). In the 4T1 “cold” model, treatment with anti–TGF-β alone or in combination with anti–PD-L1 was ineffective (Supplemental Figure 5, B and C), and, interestingly, so was the combination of anti–TGF-β plus anti–PD-L1 plus Eri or CDDP alone (as a monochemotherapy) (Supplemental Figure 5, B and C). On the other hand, when anti–TGF-β was administered with the chemotherapy combination CDDP-Eri plus anti–PD-L1, it significantly delayed tumor growth and significantly improved animal survival (Figure 5A and Supplemental Figure 5, D and E), particularly in comparison with the chemotherapy combination alone or the chemotherapy plus anti–PD-L1 combination. The efficacy of this quadritherapy was also observed in the EMT6 model (Figure 5B) as well as in the TS/A and MMTV-PyMT models (Figure 5, C and D). We have seen that the CDDP-Eri combination induced TGF-β expression in both tumor cells and some TME-associated cells (Mo-MDSCs, TAM2s, and CAFs) (Figure 4G). In order to assess the role of TGF-β1 specifically produced by tumor cells in CDDP-Eri plus anti–PD-L1 resistance, we developed a *Tgfb1*^–/–^ 4T1 cell line (Supplemental Figure 5F). We first evaluated *Tgfb1* gene induction in tumor tissue after CDDP-Eri treatment and observed that chemotherapy-induced *Tgfb1* expression was lower, but not completely lost, in *Tgfb1*^–/–^ compared with WT 4T1 tumor tissue (Supplemental Figure 5G). We then evaluated the sensitivity of WT and *Tgfb1*^–/–^ 4T1 tumor–bearing mice to chemotherapy with or without PD-L1 blockade. Interestingly, we observed significantly better therapeutic efficacy of CDDP-Eri plus anti–PD-L1 in *Tgfb1*^–/–^ 4T1 compared with WT cells, in which the chemoimmunotherapeutic effect was not different from that of chemotherapy alone. It should be noted, however, that the effect of tumor-specific depletion of TGF-β did not have as strong an effect as complete blockade of the cytokine (from tumor cells and MCT) with an anti–TGF-β antibody (Supplemental Figure 5, H and I). To assess whether this favorable effect of anti–TGF-β treatment was mediated by a positive effect on the antitumor immune response (in particular, the CTL response), we performed the same therapeutic combinations but with and without depleting anti-CD8b antibody treatment. Interestingly CTL depletion completely abolished the therapeutic effect of the quadritherapy but not CDDP-Eri plus anti–PD-L1 combination therapy, confirming the strong role of TGF-β in chemoimmunotherapy resistance in our TNBC models (Figure 5, E and F).

Thus, our results confirm the detrimental consequences of TGF-β induced by immunogenic chemotherapy with the CDDP-Eri combination. Targeting TGF-β in vivo enhanced the therapeutic efficacy of immunogenic CDDP-Eri chemotherapy in combination with anti–PD-L1, and this effect was dependent on previously recruited CTLs.

### Therapeutic targeting of TGF-β transforms the global immune context of the TNBC TME, which then becomes favorable to the efficacy of CDDP-Eri combined with anti–PD-L1.

We then sought to assess in greater detail the immunological effects of targeting TGF-β in our tumor models treated with the combination of immunogenic chemotherapy consisting of CDDP-Eri and anti–PD-L1 therapy.

**Figure 6:**
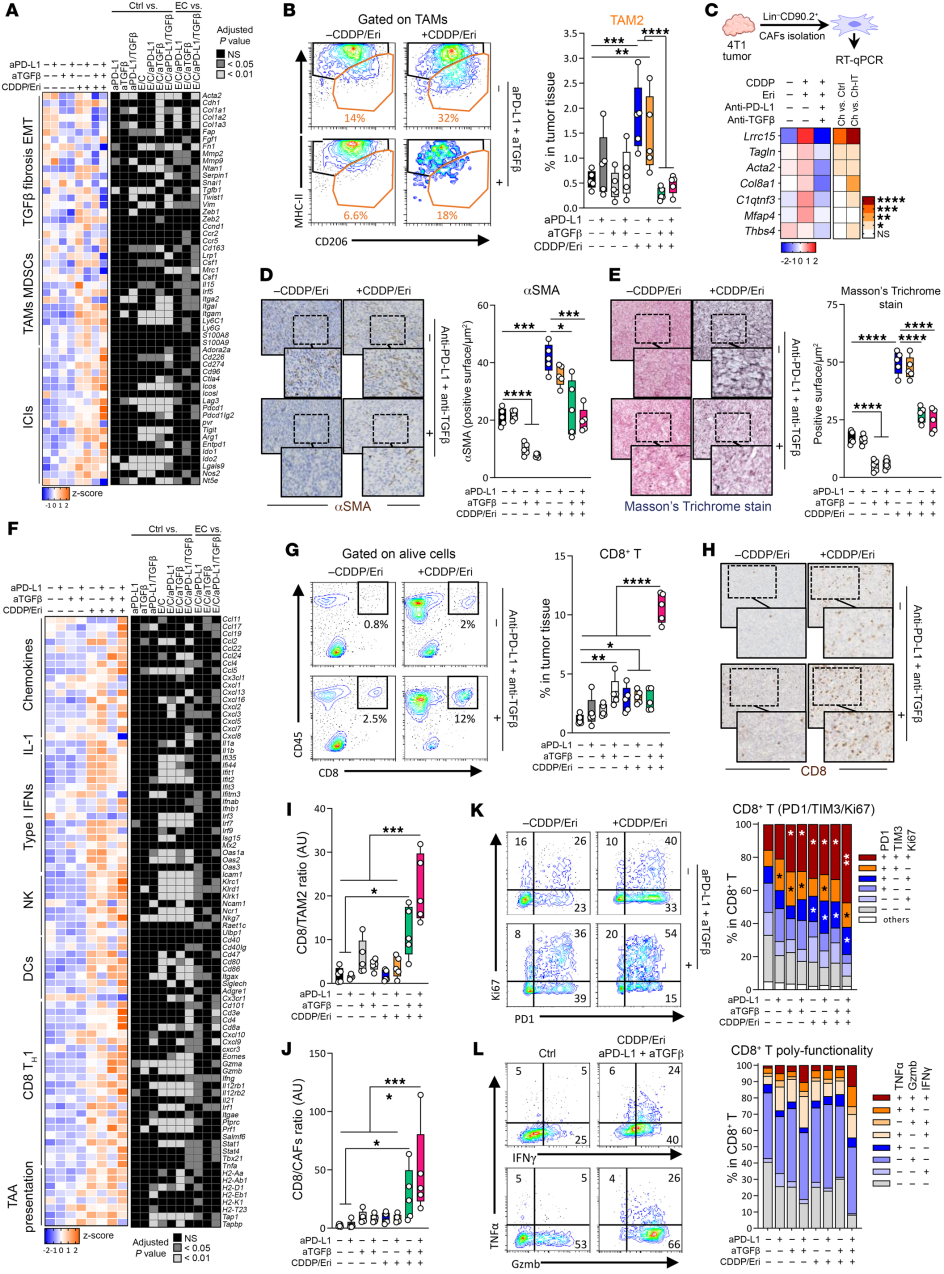
TGF-β blockade reduces immunosuppression induced by chemotherapy combination. (**A**) 4T1 tumor–bearing mice were treated with CDDP-Eri doublet, anti–TGF-β, anti–PD-L1, or a combination of these treatments. Tumors were recovered 8 days later. Total tumor mRNA was extracted, and immunosuppressive pathway gene expression was analyzed by NanoString. The heatmap on the left corresponds to normalized marker expression and the heatmap on the right indicates the *P* value from statistical analysis (at least *n* = 3 mice/group). Statistical significance was determined by 2-way ANOVA. (**B**–**L**) 4T1 tumor–bearing mice were treated as in **A**. (**B**) Proportions of TAM2s among total living cells were measured by flow cytometry. (**C**) Total CAFs were isolated using magnetic activated cell sorting (MACS), and MyCAF-related gene expression was analyzed by qPCR. (**D**) αSMA levels were measured by IHC in the tumor and automatically quantified with QPath software and (**E**) intratumoral collagen fibrosis fiber deposits were analyzed by Masson’s trichrome staining. Scale bars: 200 μm and 50 μm (enlarged insets). (**F**) Total tumor mRNA was extracted, and immune-related gene expression was analyzed by NanoString. The heatmap on the left corresponds to normalized marker expression, and the heatmap on the right indicates the *P* value from statistical analysis (2-way ANOVA). (**G**) Proportions of CTLs among total living cells were measured by flow cytometry. (**H**) CD8 was measured by IHC and automatically quantified with QPath software (scale bars: 200 μm). The CD8/TAM2 ratio (**I**) and CD8/CAF ratio (**J**) in tumors were calculated. (**K**) CD8^+^ activation was evaluated by analysis of PD-1, TIM-3, and Ki67 markers, and (**L**) the functionality of CD8^+^ was evaluated by analysis of GzmB, TNF-α, and IFN-γ expression. *n* = 5 mice/group. Data represent the mean ± SEM. *P < 0.05, **P < 0.01, ***P < 0.0001, and ****P < 0.0001, by 2-way ANOVA.

First, we assessed the effects of adding anti–TGF-β treatment on the elements of the immunosuppressive microenvironment being established under the effect of the CDDP-Eri combination, and which we have described above in our models. Via tumor transcriptome studies, we observed that the immunosuppressive pathways (TGF-β, fibrosis, EMT, TAM2-mediated immunosuppression) that were induced in 4T1 tumors after treatment with the combination of chemotherapies were downregulated by the addition of anti–TGF-β, while at the same time there was an increase in the expression of inhibitory checkpoint genes, indicative of T lymphocyte activation (Figure 6A). More specifically, flow cytometric analysis of the immune cell composition of treated tumors revealed that chemotherapy and dual PD-L1 and TGF-β blockade treatment was not accompanied by a change in the overall density of immune cells in the TME as compared with chemotherapy alone or combined with anti–PD-L1 or anti–TGF-β (Supplemental Figure 6A), but rather resulted in qualitative changes in immune infiltrates. In the case of lymphoid cells, for example, cotreatment with anti–TGF-β was accompanied by an increase in the percentage of conventional CD4^+^ T cells but, paradoxically, also of Tregs (Supplemental Figure 6B). With regard to myeloid cells, the percentage of tumor-infiltrating MDSCs was not significantly altered by quadritherapy, whether these were polymorphonuclear (PMN) MDSCs or Mo-MDSCs (Supplemental Figure 6C). In fact, the most significant variation induced by the addition of anti–TGF-β occurred in TAM2s, the percentages of which were greatly reduced (Figure 6B), without significant alteration of the total density of TAMs, TAM1s, or the TAM1/TAM2 ratio (Supplemental Figure 6, E and F). We observed the same results in the EMT6 tumor model (Supplemental Figure 7, A–F), but with a significant drop in TAM2s accompanied here by a significant increase in the TAM1/TAM2 ratio (Supplemental Figure 7, D–F). Concerning the effects on CAFs within the TME, anti–TGF-β treatment was able to decrease the percentage of intratumoral CAFs as much as with CDDP-Eri treatment, but without any significant additive or synergistic effects of the combination (Supplemental Figure 6G). In contrast, the expression of TGF-β–related genes of CAFs was completely altered by the addition of anti–TGF-β, which was able to significantly reverse the deleterious protumoral and immunosuppressive myCAFs (C0–C5 transcriptomic profile) induced by the chemotherapy combination (Figure 6C). In agreement with this observation, tumor infiltration with αSMA^+^ spindle cells (Figure 6D), as well as the tumor surface area of fibrous connective tissue (Figure 6E), was significantly reduced with anti–TGF-β treatment, particularly in combination with chemotherapy. With these immunosuppressive factors removed by anti–TGF-β treatment, the transcriptome of 4T1 tumors treated with quadritherapy showed that, compared with other therapeutic combinations, the CDDP-Eri plus anti–PD-L1 plus anti–TGF-β combination was associated with upregulation of many of the genes involved in the immune response, particularly in (a) immune cell recruitment (*Ccl5*, *Cxcl13*, *Cxcl9/10*); (b) the NK response (*Nkg7*, *Klrk1*, *Klrd1*, *Klrc1*); (c) costimulatory signals (*Cd40*, *Cd80/86*); (d) CTL response and Th1 polarization (*Cd3e*, *Cd8a*, *Cd4*, *Tnfa*, *Ifng*, *Il12rb1-2*, *Tbx21*); and (e) antigenic presentation (*H2kd*, *H2dd*) (Figure 6F). Histological examination of the treated tumors confirmed that the combination of anti–TGF-β treatment with CDDP-Eri plus anti–PD-L1 was accompanied by much greater infiltration of 4T1 tumors by CTLs (Figure 6, G and H) and, given the observations made previously, by a significantly higher CD8/TAM2 ratio (Figure 6I) and CD8/CAF ratio (Figure 6J) compared with the other treatment conditions. These CTL-infiltrated tumors expressed more proliferative and activation/exhaustion markers (Figure 6K), with a higher proportion of polyfunctional CD8^+^ TILs (Figure 6L). Interestingly, we also observed that chemotherapy combined with anti–TGF-β additively induced PD-L1 expression by tumor cells and Mo-MDSC cells (Supplemental Figure 6, H–K). All these results were also observed in the EMT6 model (Supplemental Figure 7, G–K).

Taken together, these results indicate that the TGF-β targeting induced by CDDP-Eri counteracted the broad immunosuppressive effects of this cytokine and thus restored a global immunological context within the TME, which was favorable to the efficacy of anti–PD-L1 immunotherapy combined with immunogenic chemotherapy.

### In human TNBC, TGF-β can be induced by CDDP-Eri combination treatment and confers an unfavorable prognosis, even when signs of a cytotoxic T cell response are present.

**Figure 7:**
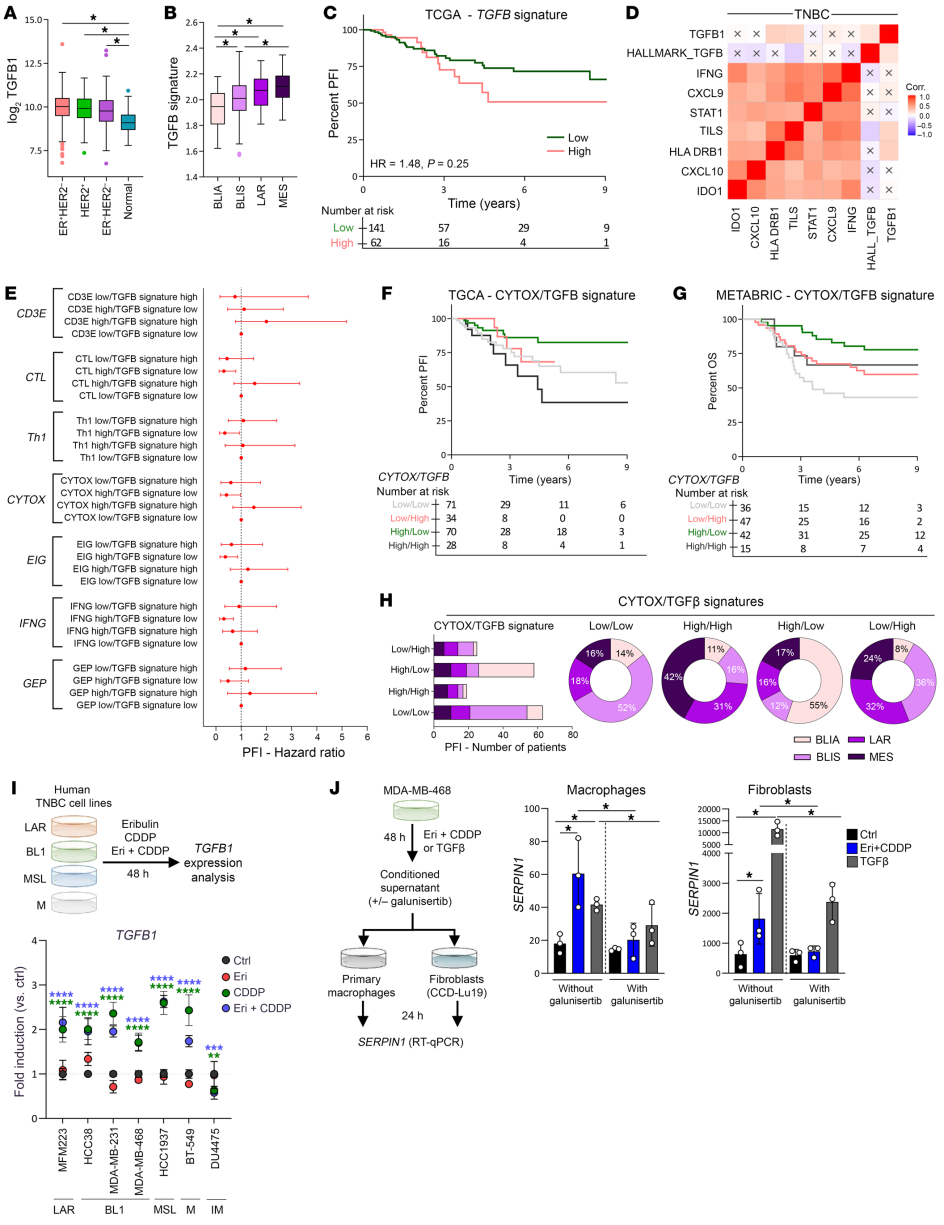
The CDDP-Eri combination induces TGF-β in human BC cell lines. (**A**) Box plots of *TGFB1* gene expression across standard pathological classifications (ER^+^HER2^–^, HER2^+^, ER^–^HER2^–^) and normal breast tissue. (**B**) *TGFB1*-related metagene expression in TNBC subtypes (BLIA, BLIS, LAR, MES) from the Burstein classification. (**C**) Kaplan-Meier curves for PFI based on *TGFB1* metagene expression. Green: low expression; red: high expression. Ticks denote censored data. (**D**) Heatmap of correlations between *TGFB1* gene, *TGFB1* metagene, and immune signatures (*IFNG*, *CXCL9*, *STAT1*, *TILs*, *HLA*, *DRB1*, *CXCL10*, *IDO1*) in TNBC. (**E**) Forest plots showing HRs for immune signatures and the *TGFB1* metagene in relation to PFI (left). (**F**) Kaplan-Meier curves for PFI based on CYTOX and *TGFB1* metagene expression in TCGA. Gray: CYTOX^lo^TGFB1^lo^; red: CYTOX^lo^TGFB1^hi^; green: CYTOX^hi^TGFB1^lo^; black: CYTOX^hi^TGFB1^hi^. (**G**) Kaplan-Meier curves for OS based on CYTOX and *TGFB1* metagene expression in METABRIC (same color coding as in **F**). (**H**) Bar and pie charts depicting the distribution of patients among TNBC subtypes (BLIA, BLIS, LAR, MES) based on *CYTOX-TGFB1* metagene combinations. (**I**) TNBC cell lines (MFM-223, HCC38, MDA-MB-231, MDA-MB-468, HCC1937, BT-549, DU4475) were treated with Eri, CDDP, or both (IC_50_ at 48 hours). *TGFB1* expression was measured by qPCR (3 independent experiments, *n* = 3/experiment). (**J**) MDA-MB-468 cells were treated with CDDP-Eri, and supernatant was applied to human macrophages and CCD-Lu19 fibroblasts pretreated with galunisertib (100 nM, 24 hours). *SERPIN1* expression was analyzed by qPCR. *P < 0.05, **P < 0.01, ***P < 0.0001, and ****P < 0.0001, by 1-way ANOVA.

In order to assess whether our results obtained in preclinical mouse models also have clinical relevance in humans, we evaluated different human public transcriptomic databases (The Cancer Genome Atlas [TCGA] and Molecular Taxonomy of Breast Cancer International Consortium [METABRIC]) to assess the prognostic effect of TGF-β (gene expression level) and TGF-β signaling (TGF-β1–associated metagene) (17) on prognosis, as well as their links to the immune response in human TNBC. This analysis revealed that the *Tgfb1* gene was significantly more highly expressed in BCs (regardless of subtype) than in normal breast tissue (Figure 7A). Among the different transcriptomic subtypes of TNBCs (18), the TGF-β1 metagene is more strongly expressed in the TNBC subtypes that are less responsive to immunotherapy (basal-like immunosuppressed [BLIS], luminal androgen receptor [LAR], and mesenchymal [MES] subtypes) compared with the basal-like, immune-activated (BLIA) subtype, which is the subtype most responsive to anti–PD-L1 therapy (4) (Figure 7B). A high level of TGF-β1–related metagene expression appears to be associated with worse prognosis in terms of progression-free interval (PFI) (Figure 7C) and overall survival (OS) (Supplemental Figure 8A) in patients with localized TNBC.

On the contrary, in localized TNBCs, signs of an antitumor immune response (such as the presence of TILs or an IFN-γ inflammatory signature [gene expression profiling (GEP)]) are classically associated with a better prognosis (19), which we found again in TCGA database, with better OS for patients with a cytotoxicity [CYTOX] tumor expression signature (20) or a highly expressed GEP signature (Supplemental Figure 8, B and C). In human TNBCs, the genes implicated in the antitumor immune response and constituting the GEP signature (*Ifng*, *Cxcl9/10*, *Stat1*) had highly correlated expression levels (Figure 7D), whereas their expression was totally uncorrelated with either *Tgfb1* or TGF-β1–related metagene expression (Figure 7D). This observation holds true, irrespective of the transcriptomic subtype of TNBC (Supplemental Figure 8D). These 2 aspects of TNBC biology therefore appear to be independent, and we therefore analyzed them jointly to estimate their mutual influence on the prognosis of patients with TNBC. In TCGA TNBC series, we examined the prognostic effect of TGF-β pathway activation (TGF-β1–associated metagene expression) in TNBC with or without evidence of a favorable antitumor immune response (expression level of various immune response signatures: *Cd3ε* (gene expression), CTL, Th1, CYTOX (20), expanded immune gene (EIG), IFN-γ, and GEP (21) (Figure 7E). We thus show that high expression of these various immune response signatures was associated with a better prognosis (PFI and OS), but only when TGF-β1 metagene expression was low (Figure 7E). For each of the immune response signatures examined, even in the case of significantly high expression, the coexistence of a highly expressed TGF-β signature (and which corresponds to the tumor profile in our models after treatment with the CDDP-Eri combination) then abolished the favorable prognostic effect. This is illustrated, for example, by the PFI curves for TNBCs according to the level of coexpression of cytotoxicity signatures (CYTOX) and the TGF-β1 metagene (Figure 7F). A similar trend was also found in an independent dataset (METABRIC), with the OS of patients with TNBC as a function of the same signatures (Figure 7G).

We then investigated whether there were differences in the coexpression profiles of these 2 signatures (TGF-β and immune response) with opposing prognostic roles, depending on the molecular subtype of human TNBC. Our analysis revealed that the different expression profiles (TGF-β1–related metagene combined with high or low CYTOX/GEP expression, respectively) could be found in each TNBC subtype (Figure 7H and Supplemental Figure 8E), but with significant differences in frequency: TNBCs with a favorable prognostic profile (immune response^hi^/TGF-β^lo^) were more frequently of the BLIA type (which in clinical settings respond better to immunotherapy), whereas subtypes with a TGF-β^hi^ signature mainly involved mesenchymal or LAR TNBCs (Figure 7H and Supplemental Figure 8E), which respond less well to immunotherapy in clinical settings (4). These results therefore show that, in humans, the immune response^hi^TGF-β^hi^ profile generated in our preclinical models by the immunogenic chemotherapy CDDP-Eri also exists in the basal state in certain TNBCs, especially of the MES subtype, with a major unfavorable prognostic effect of TGF-β.

Finally, we sought to examine whether chemotherapy with CDDP or CDDP-Eri was capable of inducing TGF-β expression in human TNBC cell lines, as in our preclinical mouse models. We therefore treated 7 different human TNBC cell lines in vitro that were representative of the different transcriptomic subtypes of this disease with Eri, CDDP, or the combination CDDP-Eri (IC_50_ is shown in Supplemental Figure 9, A–D). In 6 of our cell lines, as in our mouse models, we observed induction of *Tgfb1* gene expression when the tumor cells were treated with CDDP or its combination with Eri, irrespective of the transcriptomic subtype of TNBC (Figure 7I and Supplemental 9E). DU-4475 line, the only cell line in which this phenomenon was not observed, corresponds to an immunomodulatory subtype that is enriched for the expression of immune response genes. This chemotherapy-induced TGF-β in human cell lines was again biologically active, as we show that conditioned medium from the human MDA-MB-468 cell line previously treated with CDDP-Eri was able to induce *Serpin1* gene expression in cultured human macrophages and fibroblasts and that this was abolished by galunisertib treatment (Figure 7J).

Taken together, these analyses reveal that the CDDP-Eri combination was capable of inducing biologically active TGF-β in human TNBC cell lines and that this cytokine had a major prognostic effect in human TNBC, even when favorable signs of the immune response were present. These human data are therefore in line with our observations in preclinical models and may suggest the relevance of targeting TGF-β in the context of TNBC treated with immunogenic chemotherapy combined with anti–PD-(L)1 immunotherapy.

## Discussion

Here, we report the dual effects (from an immunological viewpoint) of an experimental platinum–based doublet chemotherapy with agents widely used in mTNBC, as well as the strategy for therapeutic associations that yield synergy with immunotherapy using an ICI in poorly immunogenic tumor models.

In our preclinical models of TNBC, the association of CDDP and Eri strongly synergized in vivo from an immunological standpoint, especially with regard to intratumoral recruitment of immune response effectors. However, this effect was limited by the concomitant induction of TGF-β, which needed to be targeted in order to enable the therapeutic synergy with an anti–PD-L1.

Only a small proportion of mTNBCs respond to immunotherapy with anti–PD-(L)1 (1). The mechanisms of this primary resistance remain to be elucidated, but it seems that immunologically inert tumors are more common at the metastatic stage than at the early stage of disease (22). These “cold” tumors (which present an immune-excluded or ignored morphotype [ref. 3], or a BLIS transcriptomic subtype [ref. 4]) are refractory to treatment with anti–PD-(L)1 agents. There is thus an important unmet clinical need for these tumors and a compelling need to identify strategies that could help to “heat up” the tumor immunologically to facilitate the action of ICIs. For this reason, among the models of TNBC, we chose an immune-ignored model associated with a strong immunosuppressive burden (4T1 model), and an immune-excluded model (EMT6).

The possibility of “heating up” poorly immunogenic cold tumors, thanks to the properties of certain chemotherapies and their effect on the immune response, has been suggested by numerous preclinical and clinical studies (6). Concerning the immunological effects of chemotherapies used to treat mTNBC, there is already a large body of published evidence surrounding the 2 main families of drugs used in routine practice, namely anthracyclines and taxanes. According to this evidence, the taxane family does not appear to have very pronounced immunological effects (23). Regarding anthracyclines, numerous publications have reported that DXR is capable of inducing the different stages of ICD (5). Anthracyclines are currently considered the standard immunogenic chemotherapy, and for this reason, we chose to use anthracyclines as the control condition in our in vitro and in vivo experiments.

CDDP and Eri, beyond their direct cytotoxic effects on tumor cells, also seem to present synergy in immunological terms. We report here, for the first time to the best of our knowledge, the results of exhaustive analyses of all the stages of ICD and tumor cell antigenicity induced by CDDP and Eri. Our findings show that CDDP was able to induce signals of ICD in TNBC cell lines and had an effect that was at least equivalent, if not superior, to that of DXR. These preclinical results are in line with observations in humans in the TONIC clinical trial (9), in which anthracyclines and CDDP were shown to be capable of inducing intratumoral signs of an immune response, thus promoting the clinical response observed after administration of an anti–PD-1 antibody. Interestingly, we show here that the CDDP-Eri association was not synergistic in vitro for the induction of various signals of ICD, and, consequently, this was not the main mechanism driving the immunological synergy that we observed in vivo. The mechanism by which Eri (which appears to be poorly immunogenic in vitro) achieved this immunological synergy with CDDP was not fully elucidated by our models.

Eri has numerous biological effects other than its cytotoxic action, notably its effects on tumor vasculature remodeling and tumor perfusion (24), which enable improved delivery of chemotherapy in some models. In our study, we also observed these antitumor vascularization effects, but noted that the antiangiogenic properties of Eri were not responsible for better penetration of CDDP into the tumors and therefore a higher concentration of intratumoral immunogenic platinum compounds. However, we found that the antiangiogenic effects of Eri were significantly associated with CTL infiltration, and we could speculate that Eri facilitated CTL tumor trafficking. Similarly, although it has been suggested that part of the antitumor effect of Eri may be linked to a role countering the EMT state, possibly via downregulation of the TGF-β/Smad pathway (25), we observed no modulating effect of Eri on the TGF-β pathway in the TNBC models tested in our study.

Our results show that in murine models of TNBC, as well as in human cell lines, treatment with CDDP induced strong tumor production of TGF-β by a wide range of different cell populations from the TME. The possibility of increased TGF-β signaling after chemotherapy had previously been suggested in the setting of TNBC after treatment with taxanes, and this condition could promote the expansion of cancer stem-like cells (26). Other chemotherapy drugs such as anthracyclines also seem capable of inducing TGF-β production in various cancer models (27), as can radiotherapy (28). In vitro, in cervical and ovarian cancer cell lines, CDDP induces autocrine TGF-β signaling (29). In these studies, the deleterious effect of chemotherapy-induced TGF-β signaling was mediated by a change in tumor cell phenotype (enhancing cancer stem-like cell properties and EMT), thereby rendering them resistant to chemotherapy (29). Our study is therefore the first to our knowledge to show that chemotherapy-induced TGF-β signaling may also contribute to resistance to immunotherapy in the therapeutic context of an ICI combined with chemotherapy.

Moreover, we describe here the profound qualitative and quantitative changes in the TME, driven by CDDP-induced TGF-β, that favored the emergence of different subpopulations of immune cells and CAFs characterized by immunosuppressive properties. It is now well recognized that CAFs play a key part in shaping the TME and response to cancer immunotherapy (30), and there is a strong relationship between CAF abundance and lack of response to anti–PD-(L)1 in the clinical setting (31). In our work, we describe for the first time to our knowledge that when chemotherapy was able to induce TGF-β in the TME, CAFs isolated from the tumors showed a more pronounced transcriptomic profile of LRRC15^+^ CAFs, a myofibroblast subset of CAFs (myCAFs). This CAF subpopulation belonging to TGF-β–induced CAF clusters (C0–C5 clusters) (15) has immunosuppressive properties (12) and has recently been described to be in abundance in BCs (14). Thus, the biological phenomena induced in CAFs by CDDP treatment in our models appears complex, with both a depleting effect on the overall CAF population, but also the emergence of a TGF-β–responsive and immunosuppressive subpopulation.

Our results show that targeting TGF-β with a specific antibody made it possible to substantially enhance the intratumoral immune response, particularly the stigmata of cytotoxicity. This has already been shown in the immuno-excluded EMT6 model, in which TGF-β appears to work with PD-L1 to prevent intratumoral stem cell–like CTL expansion and replacement of exhausted CTLs, thus maintaining the T cell compartment in a dysfunctional state (32). From a translational research standpoint, it would be of paramount importance to conduct future studies to identify which other chemotherapy molecules could prompt TGF-β production by tumors, since there are now myriad pharmacological approaches that could target this pathway. Indeed, several molecules targeting TGF-β are currently in preclinical and clinical development (33) in the form of either small molecules that block TGFBR1 activity or monoclonal antibodies or fusion proteins that block the cytokine or its activation. Some of these anti–TGF-β therapies are being developed in association with ICI immunotherapy and/or classical anticancer treatments such as radiotherapy or chemotherapy (33). Newer molecules, such as bintrafusp α, combine targets in a bifunctional fusion protein composed of the extracellular domain of the TGF-βRII receptor designed to function as a TGF-β “trap,” fused to a human IgG1 antibody blocking PD-L1 (34). Interestingly, a phase I trial is currently testing bintrafusp α in mTNBC in combination with Eri (NCT03579472). The clinical results of this trial, particularly regarding efficacy and safety, will be of interest to fuel future debate about the possibility of adding CDDP to this combination, on the basis of our preclinical findings.

As for the possibility of extrapolating our results to the clinical setting, phase I/II clinical trials have shown that Eri plus CDDP (35) is a feasible combination that does not cause excess toxicity and yields responses in diverse types of cancer, including BC. In mTNBC, the phase Ib/II ENHANCE trial (36) tested a combination of Eri plus pembrolizumab (anti–PD-1 agent) in patients who had received up to 2 prior systemic anticancer therapies in the metastatic setting and reported that the combination was generally well tolerated in terms of toxicity. Furthermore, clinical responses were observed, especially for patients whose tumors were probably more inflamed and expressed PD-L1. The same combination of Eri plus pembrolizumab was also tested in patients with metastatic, estrogen receptor–positive (ER^+^) BC (which are mainly cold tumors) (10) in a phase II trial (37) that showed no additional benefit of adding pembrolizumab compared with Eri alone. The results of these different clinical trials indicate that Eri alone is probably insufficient to achieve immunological synergy with an ICI in poorly immunogenic tumors and that the combination of CDDP plus Eri plus an ICI is likely possible in the clinical setting.

Despite being limited to human TNBC cell lines and data coming from TCGA, the translational studies performed in our work reveal the unfavorable prognostic effect of the TGF-β pathway on the immune response in human TNBC, as well as the ability of CDDP to induce this cytokine also in human cell lines. Our findings highlight the clinical relevance of targeting TGF-β in the context of human TNBC treated with chemoimmunotherapy.

Taken together, we believe our results provide a solid rationale to develop co-targeting of PD-L1 and TGF-β, in association with an efficient CDDP-Eri cytotoxic and immunogenic chemotherapy doublet, in the context of refractory/relapsing mTNBC with an unfavorable immunophenotype.

## Methods

### Sex as a biological variable.

Our study exclusively examined female mice because the disease modeled (BC) is more prevalent in females.

### Mouse strains.

All mice used in the experiments were between 6 and 8 weeks of age and were housed under standard conditions in the animal research facility (Université de Bourgogne Europe). Female BALB/c and nude NMRI mice (nu/nu) between 7 and 9 weeks of age were purchased from Charles River Laboratories. Animals were grouped randomly for each experiment.

### Mouse cell lines.

4T1 and EMT6 murine breast carcinoma cells were cultured at 37°C under 5% CO_2_ in RPMI with 10% (vol/vol) FCS (Dutscher) supplemented with penicillin and streptomycin (Gibco, Thermo Fisher Scientific). TS/A murine BC cells were cultured at 37°C under 5% CO_2_ in DMEM with 10% (vol/vol) FCS (Dutscher) supplemented with penicillin and streptomycin (Gibco, Thermo Fisher Scientific). 4T1 and EMT6 cells were obtained from the American Type Culture Collection (ATCC). TS/A cells were obtained from Merck Millipore.

Mouse lung endothelial cells (MLECs) were cultured at 37°C under 5% CO_2_ in DMEM with 10% (vol/vol) FCS (Dutscher) supplemented with penicillin and streptomycin (Gibco, Thermo Fisher Scientific). MLECs were a gift from Martin Kolb (Department of Medicine, McMaster University, Hamilton, Ontario, Canada). Cells were routinely tested for mycoplasma contamination using the Mycoalert Mycoplasma Detection Kit (Lonza).

### Human cell lines.

MDA-MB-231, MDA-MB-468, and MFM-223 human BC cells were cultured at 37°C under 5% CO_2_ in DMEM with 10% (vol/vol) FCS supplemented with penicillin and streptomycin. HCC38, HCC1937, and DU4475 human BC cells were cultured at 37°C under 5% CO_2_ in RPMI with 10% (vol/vol) FCS supplemented with penicillin and streptomycin. BT-549 human BC cells were cultured at 37°C under 5% CO_2_ in RPMI with 10% (vol/vol) FCS supplemented with penicillin and streptomycin and 1% (vol/vol) human insulin (Santa Cruz Biotechnology). MDA-MB-231, MDA-MB-468, HCC38, HCC1937, DU4475, and BT-549 were obtained from the ATCC. MFM-223 cells were obtained from Merck Millipore. CCD-19Lu fibroblastic human cells were cultured at 37°C under 5% CO_2_ in MEM with 10% (vol/vol) FCS supplemented with penicillin and streptomycin. CCD19Lu was obtained from the ATCC. Cells were routinely tested for mycoplasma contamination using the Mycoalert Mycoplasma Detection Kit.

### Real-time quantitative PCR.

Murine cell lines were seeded in 24-well plates at 50,000 cells/well (4T1) and 28,000 cells/well (EMT6) the day before treatment with chemotherapies. Human cell lines were seeded in 24-well plates at 112,000 cells/well (MDA-MB-231 and MDA-MB-468), 36,000 cells/well (HCC38), 55,000 cells/well (HCC1937), 70,000 cells/well (BT-549 and MFM-223), and 140,000 cells/well (DU4475). Total RNA from tumor cells was extracted with TriReagent (Thermo Fisher Scientific) and assayed with NanoDrop (Thermo Fisher Scientific). RNA (300 ng) was reverse-transcribed using M-MLV reverse transcriptase (Invitrogen, Thermo Fisher Scientific). The cDNAs obtained were analyzed by real-time quantitative PCR (qPCR) with SYBR green (Thermo Fisher Scientific) using the QuantStudio 5 Real-time PCR system (Applied Biosystems). Expression was normalized to the expression of mouse or human *Actb/ACTB*. The primers used are available in Supplemental Table 2.

### nCounter gene expression analysis.

Total RNA (100 ng) was used in the nCounter assay (NanoString Technologies) using our mouse-specific nCounter custom panel following the manufacturer’s recommendations. Samples were prepared using an nCounter Prep Station, and the code set and RNA complexes were immobilized on nCounter cartridges for data collection. Data were collected on a NanoString Digital Analyzer (NanoString Technologies). nCounter RNA count data were normalized using the geometric mean of the positive controls and housekeeping genes. In this study, the nCounter panel included 183 genes (see Supplemental Table 3 for gene and probe information). Normalization was performed using *z* score calculations, and ANOVA was used for statistical analysis.

### Syngeneic transplantable tumor models.

Tumor formation was induced by orthotopic injections of 1.10^5^ 4T1, EMT6, or TS/A cells into BALB/c mice or nude NMRI mice. Mice were randomized and treated between 8 and 10 days after tumor cell implantation.

### In vivo treatments.

For in vivo WT 4T1 or 4T1 *Tgfb1^–/–^*, EMT6, or TS/A tumor growth experiments, Eri (1 mg/kg) or NaCl 0.9% was administered by i.v. injection on the day of randomization and 4 days after the primary injection. CDDP (6 mg/kg) or NaCl 0.9% was administered once by i.p. injection at randomization. Anti–PD-L1 (10 mg/kg; BE0101, B7-H1), anti–TGF-β (10 mg/kg; BE0057, 1D11.16.8) antibodies or isotype control antibodies (all from BioXCell) were injected i.p. at a dose of 10 mg/kg twice a week for 3 weeks.

### CD8b depletion.

For in vivo 4T1 and EMT6 tumor growth experiments, anti-CD8a (BE0061 2.43) or an isotype control antibody (both from BioXCell) were administered by i.p. injection at a dose of 10 mg/kg one day before randomization, then twice a week for 3 weeks after the primary injection.

### Flow cytometry.

To study the infiltration of lymphoid and myeloid cells in the tumor tissue, tumors were collected 8 days after randomization and treatment. After dissection, the tumors were mechanically and enzymatically dissociated using a mouse tumor dissociation kit according to the manufacturer’s recommendations (Miltenyi Biotec). Myeloid cell infiltration was analyzed by staining the tumor cell suspension (10^6^ cells) in Flow Cytometry Staining Buffer (FSB) (eBioscience) with specific antibodies according to the manufacturer’s recommendations for 15 minutes at room temperature in the dark and then washing the cell suspension twice in FSB followed by flow cytometric analysis.

Infiltration of lymphoid cells and their exhaustion were analyzed by staining of surface markers. Cells were then fixed and permeabilized with a Foxp3 staining buffer set, according to the manufacturer’s instructions (Miltenyi Biotec), and intracellular proteins were stained.

To study the cytokine function of the lymphoid infiltrate, PMA (20 ng/mL; MilliporeSigma), ionomycin (1 μg/mL; MilliporeSigma), and brefeldin A (2 μL/mL; eBioscience) were added in the tumor cell suspension for 3 hours at 37°C. After staining for surface markers, cells were fixed and permeabilized with the Foxp3 staining buffer set, according to the manufacturer’s instructions (Miltenyi Biotec), and intracellular proteins were stained. For the myeloid and lymphoid cell infiltration and function assay, the viability dye eFluor 780 (eBioscience, Thermo Fisher Scientific) was used to identify live cells.

To study the infiltration of fibroblast-associated cancer and endothelial cells in the tumor tissue, tumor cell suspension was analyzed by staining in Flow Cytometry Staining Buffer (FSB, eBioscience) with specific antibodies according to the manufacturer’s recommendations for 15 minutes at room temperature in the dark, washed twice in FSB, and analyzed by flow cytometry. Flow cytometry acquisition was performed on a Cytoflex 13C cytometer (Beckman Coulter). CytExpert (Beckman Coulter) was used for analysis.

Further details on methods can be found in the Supplemental Methods.

### Statistics.

Statistical analyses were performed using GraphPad Prism (GraphPad Software)and R software. For in vitro experiments, results are shown as the median ± IQR. Data sets were compared using an unpaired Mann-Whitney *U* Wilcoxon test. Correlations were estimated through Pearson’s correlation coefficient. *P* values of 0.05 or less were considered statistically significant. Data in the figures are presented as the mean ± SEM. For in vivo experiments, survival probabilities were estimated using the Kaplan-Meier method, and survival curves were compared using the log-rank test. The prognostic value of the different variables was tested using univariate Cox regression models for OS and PFS. Transcriptomic signatures (continuous variables) were dichotomized on the basis of the cut-off value determined using the maximally selected rank statistics of Hothorn et al. via the maxstat R library (38).

All other analyses were performed using 1-or 2-way ANOVA followed by the Šidák’s post test for multiple comparisons. Statistical analyses were performed using R software, version 4.0.3 (http://www.R-project.org/), and graphs were drawn using GraphPad Prism, version 9.0.2.

### Study approval.

All mouse protocols and experiments were performed in accordance with the Federation of European Laboratory Animal Science Associations (FELASA) and after approval by the Animal Experimental Ethics Committee (no. C 21 464 04 EA, Université de Bourgogne Europe.

### Data availability.

Values for all data points are available in the Supporting Data Values file.

## Author contributions

VD, MR, and CT contributed data or analysis tools. LK, FL, SP, CP, SKM, LG, AI, RA, and DR performed experiments and analysis. LK, FL, EL, FG, and SL conceived and designed the analysis. PSB and MT performed experiments and analysis. LK, EL, and SL wrote the manuscript.

## Supplementary Material

Supplemental data

Unedited blot and gel images

Supporting data values

## Version 1 07/01/2025

Electronic publication
