# Supplementary material for: Co-targeting TGF-β and PD-L1 sensitizes triple-negative breast cancer to experimental immunogenic cisplatin-eribulin chemotherapy doublet
Source: J Clin Invest. 2025 Jul 1;135(13):e184422. doi: 10.1172/JCI184422 (PMC12208543; doi:10.1172/JCI184422)
Supplement: Supplemental data [file jci-135-184422-s230.pdf]

Supplemental information

Supplementary Figures Legend:

Supplementary Figure 1:

A. 4T1, EMT6, and MC38 tumor-bearing mice were treated with anti-PD-L1 antibodies twice a week for two weeks, then once. Tumor volume was monitored for 30–50 days after treatment (at least n = 3 mice/group, graph represents the mean ± SEM, two-way ANOVA test).

B. 4T1 and EMT6 cells were orthotopically inoculated in BALB/c mice, and MC38 cells were inoculated (*c*) in C57BL/6 mice. Seven days later, tumors were recovered. CD8 cells were studied by IHC and automatically quantified with QPath software (scale bar, 2 mm). On the left, images show the tumor core, and on the right, images represent the peripheral tumor.

C. Proportions of CD8+ cells among total living cells were measured by flow cytometry.

D. PD-L1 positive cells were quantified by flow cytometry. On the left, the graph represents the mean ± standard deviation, and on the right, a representative dot plot is shown (n = at least 5 mice per group, two-way ANOVA test). *p < 0.05, **p < 0.01, ***p < 0.001, and ****p < 0.0001.

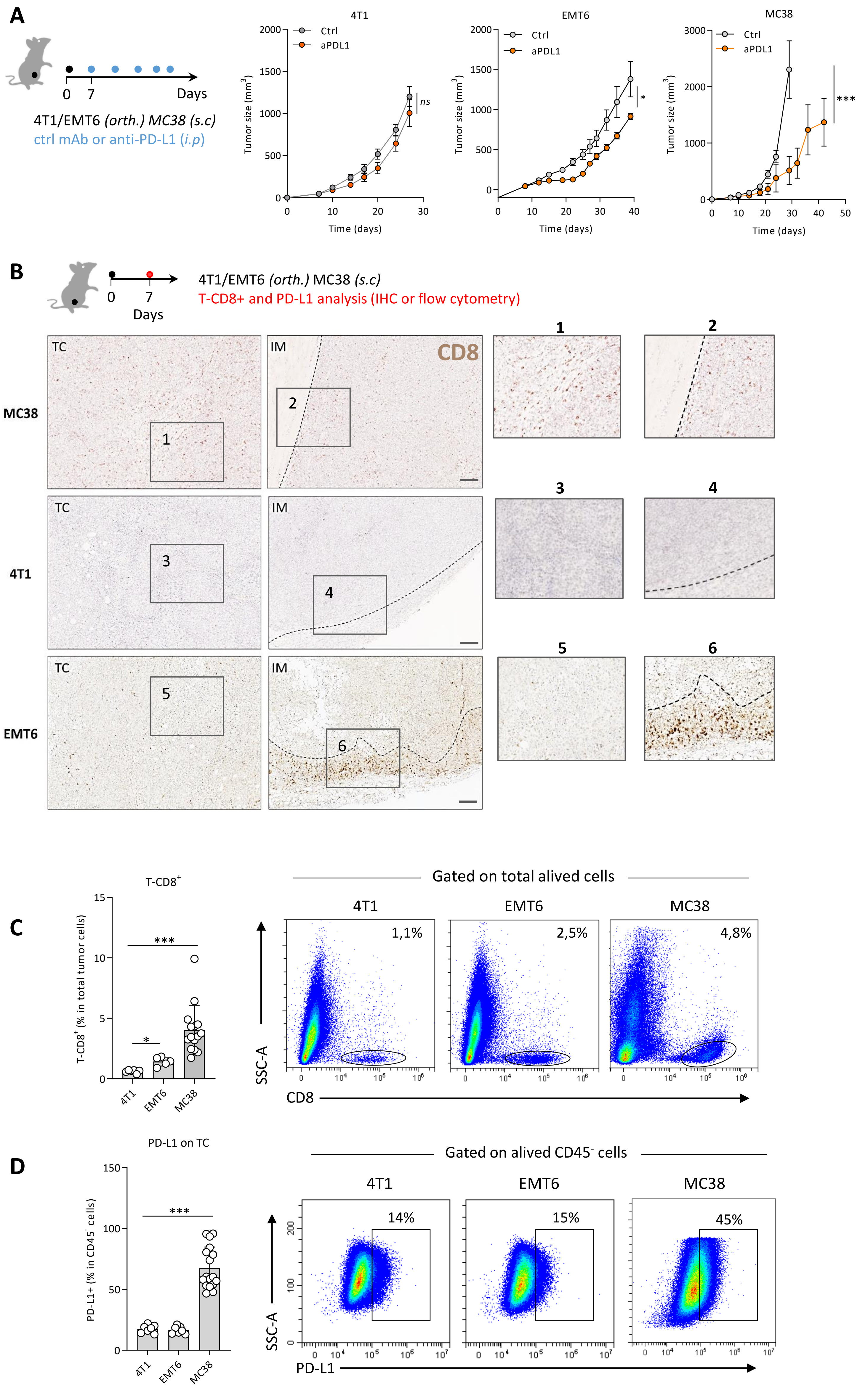

Supplementary Figure 1

Supplementary Figure 2:

A-C. 4T1 and EMT6 tumor-bearing mice were treated with CDDP, Eri, or both

A. The toxicity of these molecules was studied by monitoring the weight of mice for 30 days, box plot represents the median and interquartile range (at least n = 10 mice/group).

B. In the same conditions as A, tumors were recovered eight days after treatment. Total tumor mRNA recovered after eight days was extracted, and immunosuppressive pathway gene expression was analyzed by nanostring. The volcano plot indicates the *p*-value from the statistical analysis.

C. The distribution of CD8 cells was studied by IHC, with samples taken from the tumor center (inner) and periphery (outer) (scale bar, 2 mm) (data shown in figure 1.C).

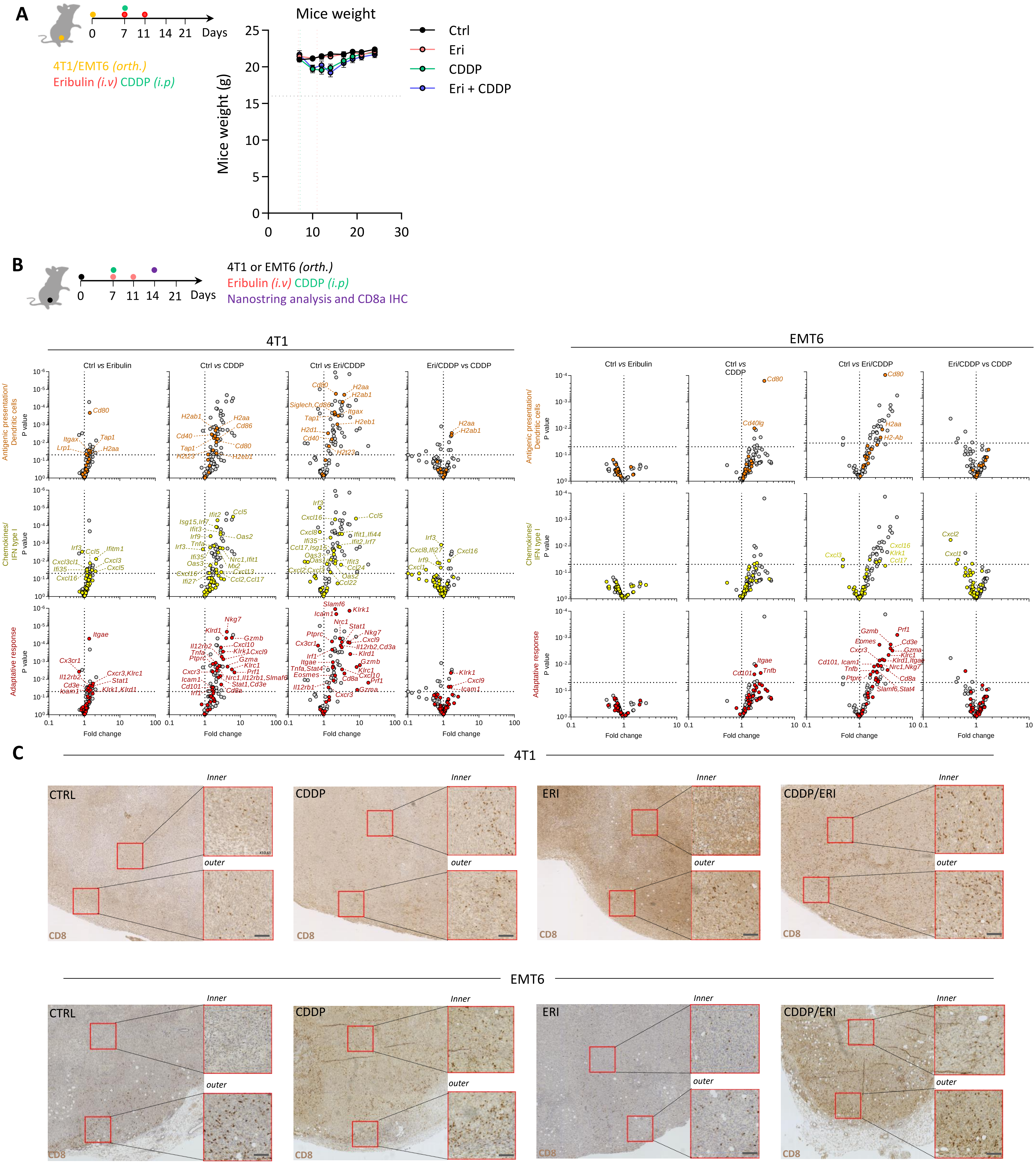

Supplementary Figure 2

Supplementary Figure 3:

A-D. 4T1 (**A**) and EMT6 (**D**) cells were treated with various doses of CDDP, Eri, and DXR. Forty-eight hours after treatment, the half-maximal inhibitory concentration (IC50) was determined by the analysis of cell viability by MTT (3 independent experiments, n = 3/experiments, one-way ANOVA test).

B-E.4T1 (**B**) and EMT6 (**E**) were treated with various doses of cisplatin (CDDP: 0, 1, 2, 4, 8, or 16 µM) and eribulin (Eri: 0, 3.9, 7.8, 15.6, 31.3, 62.5, 125, 250, 500, or 1000 nM) in combination for 48 h. Viability was assessed by an MTT assay. The heatmap represents the MTT results, showing the mean percentage of adherent cells for each condition from three independent experiments.

C-F. The proportion of cells in apoptosis and necrosis was studied by flow cytometry for 4T1 cells (**C**) and EMT6 cells (**F**) (2 independent experiments, n = 3/experiments, one-way ANOVA test).

G. EMT6 cells were treated with Eri (90 nM), CDDP (4 µM), DXR (400 nM), and a combination of CDDP with Eri or left untreated, Twenty-four and forty-eight hours after treatment, ICD markers were analyzed. The heatmap on the top corresponds to normalized marker expression, and on the bottom is the p value from statistical analysis. Pie charts on the right indicate the proportion of positive ICD markers (3 independent experiments, n = 3/experiments, one-way ANOVA test).

H. In the same conditions, Eif2α phosphorylation (Ser51), and LC3II/I were analyzed by western blot. The heatmap on the bottom represents the densitometric ratio between phosphoprotein and total protein (1 representative experiment of 2 by western blotting).

I. 4T1-bearing (orth.) mice received CDDP, Eri, or both. Four days later, tumors were recovered and CAF cells were sorted by magnetic beads, and the positive fraction was validated by the study of the expression of CAF markers by flow cytometry.

J. 4T1-bearing (orth.) mice received CDDP, Eri, or both under the same conditions as I. Six hours, one day, two days, four days, and eight days later, tumors were recovered, and platinum atoms in the tumors were quantified by inductively coupled plasma mass spectrometry (ICP-MS) n=3 mice/group. *p < 0.05, **p < 0.01, ***p < 0.001, and ****p < 0.0001.

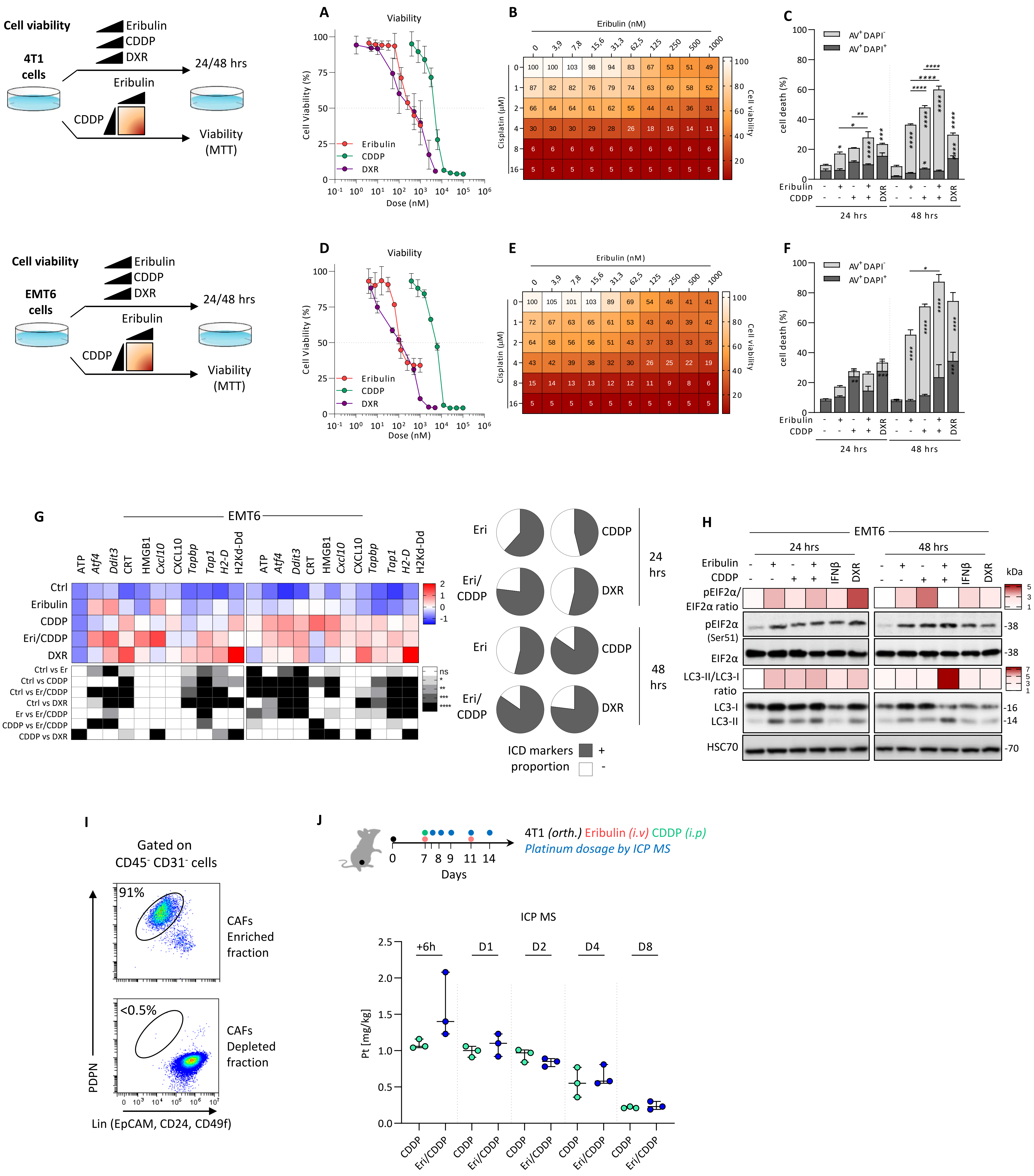

Supplementary Figure 3

Supplementary Figure 4:

A.EMT6-tumor-bearing mice were treated with CDDP, Eri, or both. Tumors were recovered four, eight, and fourteen days later. Total tumor mRNA recovered after eight days was extracted, and *Tgb1* expression was analyzed by RT-qPCR (at least n = 6 mice/group, two-way ANOVA test).

B. EMT6 cells were treated with Eri (90 nM), CDDP (4 µM), a combination of CDDP with Eri, or left untreated. Twenty-four or forty-eight hours after treatment, *Tgfb1* expression were analyzed by RT-qPCR (2 independent experiments, n = 3/experiments, two-way ANOVA test).

C. EMT6 cells were treated with Eri (90 nM), CDDP (4 µM), a combination of CDDP with Eri, or left untreated. Twenty-four or forty-eight hours after treatment, *Serpin1* expression were analyzed by RT-qPCR (2 independent experiments, n = 3/experiments, two-way ANOVA test).

D. EMT6 cells were treated by various doses of galunisertib (10; 100; and 1000 nM) for two hours, then by CDDP (4µM), TGFβ (2ng/mL), or left untreated for forty-eight hours. *Serpin1* expression was analyzed by RT-qPCR (graph represents mean +/- SEM, n = 3/experiment, one-way ANOVA test).

E-F. 4T1 cells were treated with Eri (90 nM), CDDP (4 µM), a combination of CDDP with Eri, or left untreated. Twenty-four or forty-eight hours after treatment. Expressions of *Tgfb1, Tgfb2, Tgfb3* were analyzed on 4T1 cells left untreated (**C**) or treated by chemotherapies (**D**) by RT-qPCR (2 independent experiments, n = 3/experiments, two-way ANOVA test).

G. 4T1 cells were treated with CDDP (4µM) and TGFβ (2ng/mL). Twenty-four hours after treatment, the supernatant was recovered and deposited on MLEC reporter cells. The TGFβ amount was evaluated by lysis of MLEC cells and quantification of the luciferase activity (n = 3/experiments, two-way ANOVA test).

H. 4T1 cells were treated with CDDP (4 µM) and carboplatin (64 µM), and *Tgfb1* expression was analyzed by RT-qPCR (the graph represents the mean +/- SEM, 2 independent experiments, n = 3-4/experiments, one-way ANOVA test).

I. At day eight after treatment, intratumoral collagen fibrosis was analyzed by Masson's Trichrome staining and CD8 cells were studied by IHC (scale bar, 2 mm) (data shown in figure 4.K).

J. 4T1-tumor-bearing mice were treated with CDDP, Eri, or both. Tumors were recovered four, eight, and fourteen days later, and proportions of PMN-MDSC cells among total live cells were measured by flow cytometry (at least n=4 mice/group, box show mean +/- SEM, two way ANOVA test).

K. EMT6-tumor-bearing mice were treated with CDDP, Eri, or both. Tumors were recovered four, eight, and fourteen days later, and the and the proportion of Treg, Mo-MDSCs, TAM2, and PMN-MDSC among total living cells was measured by flow cytometry (at least n = 3 mice/group, boxes show mean ± SEM, two way ANOVA test). *p < 0.05, **p < 0.01, ***p < 0.001, and ****p < 0.0001.

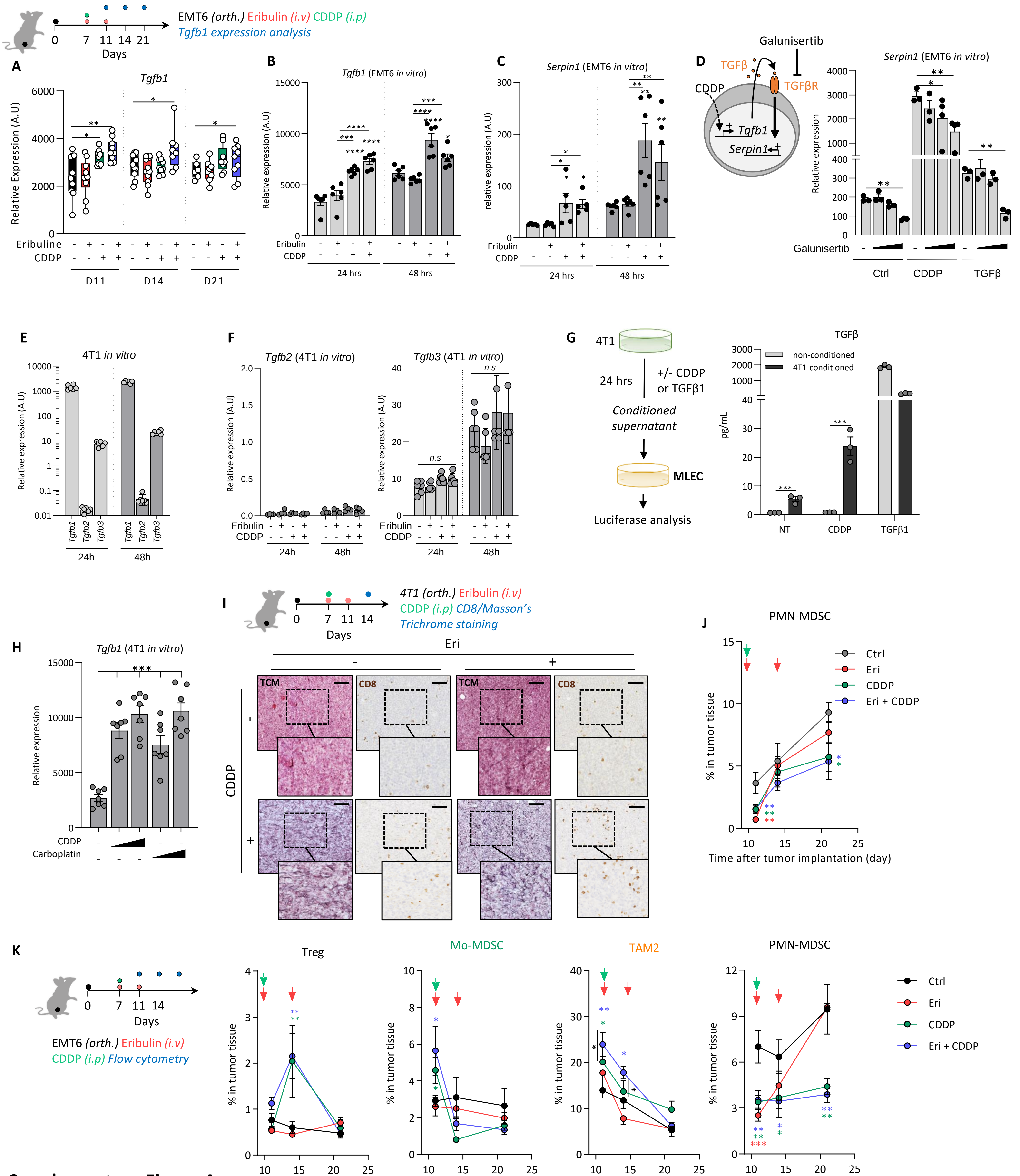

Supplementary Figure 4

Supplementary Figure 5:

A. 4T1 tumor-bearing mice were treated with CDDP/Eri doublet, anti-PD-L1/anti-TGFβ doublet, or the combination of the four molecules, or left untreated. The weight of mice was measured for 45 days, box plot represents the median and interquartile range, at least n=6 mice/group.

B-D. On the left, tumor volume was monitored; data represent mean ± SEM. On the right, mouse survival was evaluated (n = at least 5 mice/group), data represent the median, (log-rank test).

B.4T1 tumor-bearing mice were treated with Eri, anti-PD-L1, anti-TGFβ, or the combination, or left untreated.

C.4T1 tumor-bearing mice were treated with CDDP, anti-PD-L1, anti-TGFβ in monotherapy or in combination, or left untreated.

D.4T1 tumor-bearing mice were treated with Eri/CDDP doublet, anti-PD-L1, anti-TGFβ in monotherapy or in combination with chemotherapies doublet, or left untreated.

E. Tumor volume was monitored for 40 days after treatment, and mouse survival was evaluated for 50 days. The median survival of mice in each group is presented.

F. TGFβ1 secretion level was evaluated in culture supernatant derived from 4T1 WT and *Tgfb1*$^{-/-}$ cell lines. Two *Tgfb1*$^{-/-}$ clones were tested (R1 and R2).

G. *Tgfb1* expression was analyzed by RT-qPCR (from total mRNA) from 4T1 WT or *Tgfb1*$^{-/-}$ (R1 clone) tumor tissue and recovered after 4 days of treatment by CDDP/Eri. At least n = 6 mice/group, two-way ANOVA test.

H-J. WT 4T1 and *Tgfb1*$^{-/-}$ (R1 clone) tumor-bearing mice were treated with CDDP/Eri doublet, anti-PD-L1, or the combination or left untreated. Tumor volume was monitored during at least 36 days. Data represent mean ± SEM. At least n=6 mice/group.

*p < 0.05, **p < 0.01, ***p < 0.001, and ****p < 0.0001.

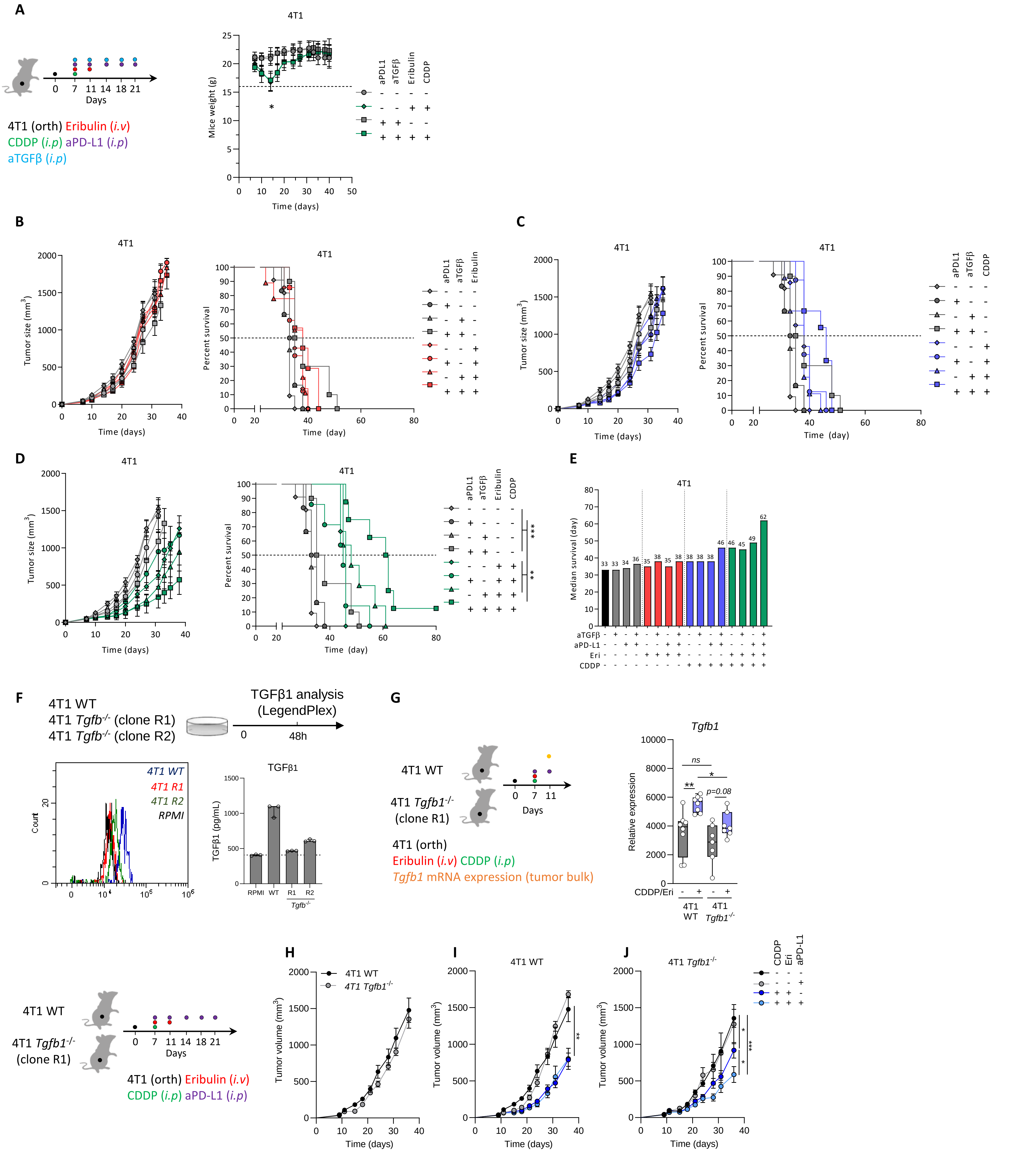

Supplementary Figure 5

Supplementary Figure 6:

A-G. 4T1 tumor-bearing mice were treated with Eri/CDDP doublet, anti-PD-L1, anti-TGFβ in monotherapy or in combination with chemotherapies doublet, or left untreated. Tumors were recovered eight days later, and proportions of CD45 TILs (**A**), T-CD4+ (**B**), PMN-MDSC (**C**), mo-MDSC (**D**), total TAMs and TAM1 (**E**), TAM1/TAM2 ratio (**F**) and total CAFs (**G**) among total lives cells were measured by flow cytometry.

H. 4T1 tumor-bearing mice were treated as in A. PD-L1 staining was analyzed on CD45- cells (tumor cells) and on Mo-MDSC both in control and anti-PD-L1 group.

I-K. 4T1 tumor-bearing mice were treated as in A. PD-L1 expression (percentage (**I**) and normalized MFI (**J**) were analyzed on CD45- cells (tumor cells) and myeloid cell subsets. Representative contour plot were shown (**K**).

(at least n = 4 mice/group; box shows median ± interquartile range, two-way ANOVA test). *p < 0.05, **p < 0.01, ***p < 0.001, and ****p < 0.0001.

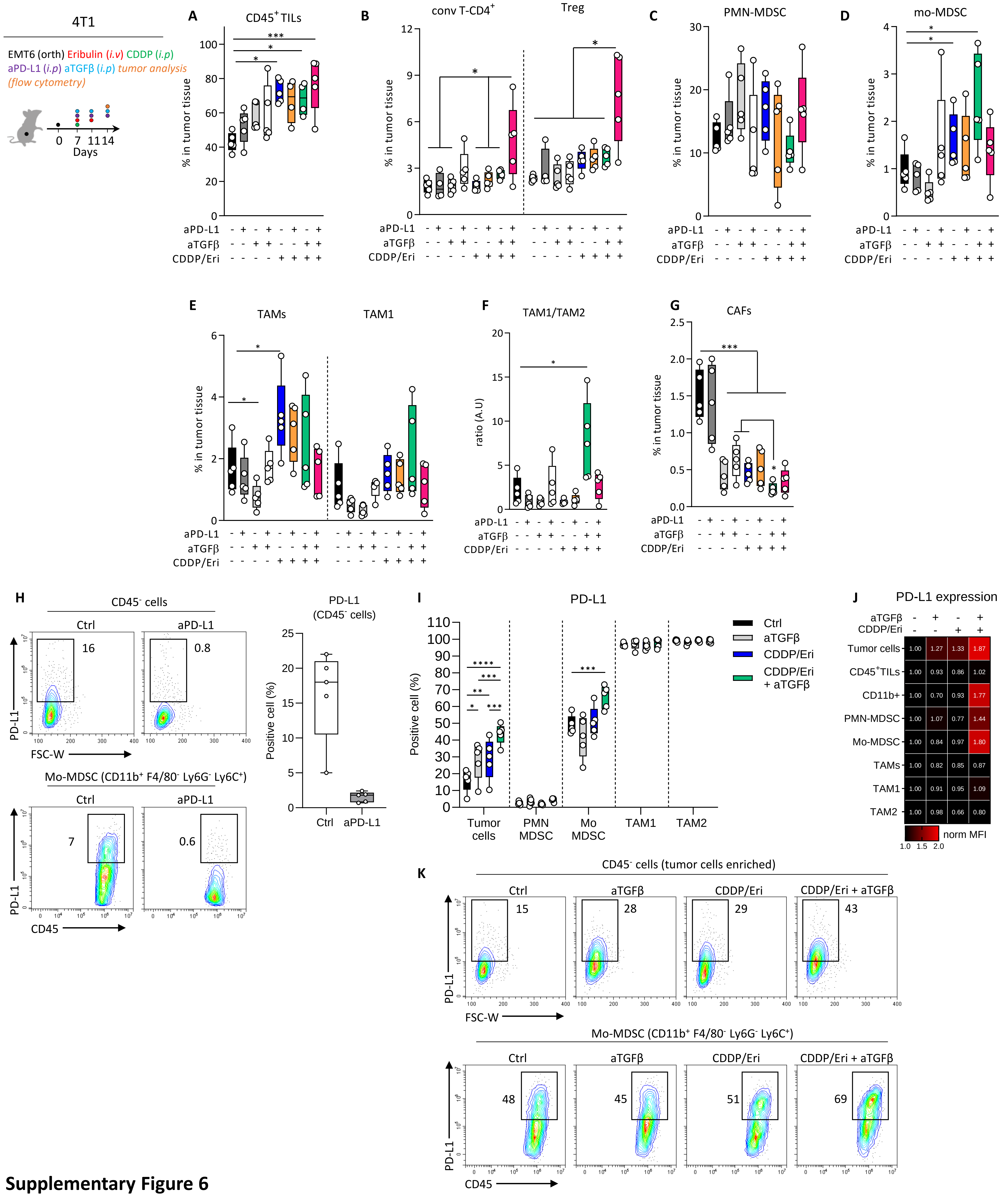

Supplementary Figure 6

Supplementary Figure 7:

A-E. EMT6 tumor-bearing mice were treated with Eri/CDDP doublet, anti-PD-L1, anti-TGFβ, or a combination of the four molecules, or left untreated. Tumors were recovered eight days later, and proportions of CD45 TILs (**A**), T-CD4+/Treg (**B**), PMN-MDSC/mo-MDSC (**C**), and TAM (**D**/**E**) among total lives cells were measured by flow cytometry.

F. The ratio of the number of TAM1 to the number of TAM2 in the tumor was evaluated.

G.CD8 was studied by IHC and automatically quantified with QPath software (scale bar, 200µm).

H. The proportion of CD8+ cells among total living cells was measured by flow cytometry.

I. The ratio of the number of CD8 cells to the number of TAM2 cells in tumors was evaluated.

J. Exhaustion of CD8+ was evaluated by analysis of PD-1, TIM-3, and Ki67 markers.

K. Functionality of CD8+ was evaluated by analysis of GzmB, TNFα, and IFNγ markers.

(n = 5 mice/group, boxes show mean ± SEM, two-way ANOVA test). *$p < 0.05$, **$p < 0.01$, ***$p < 0.001$, and ****$p < 0.0001$.

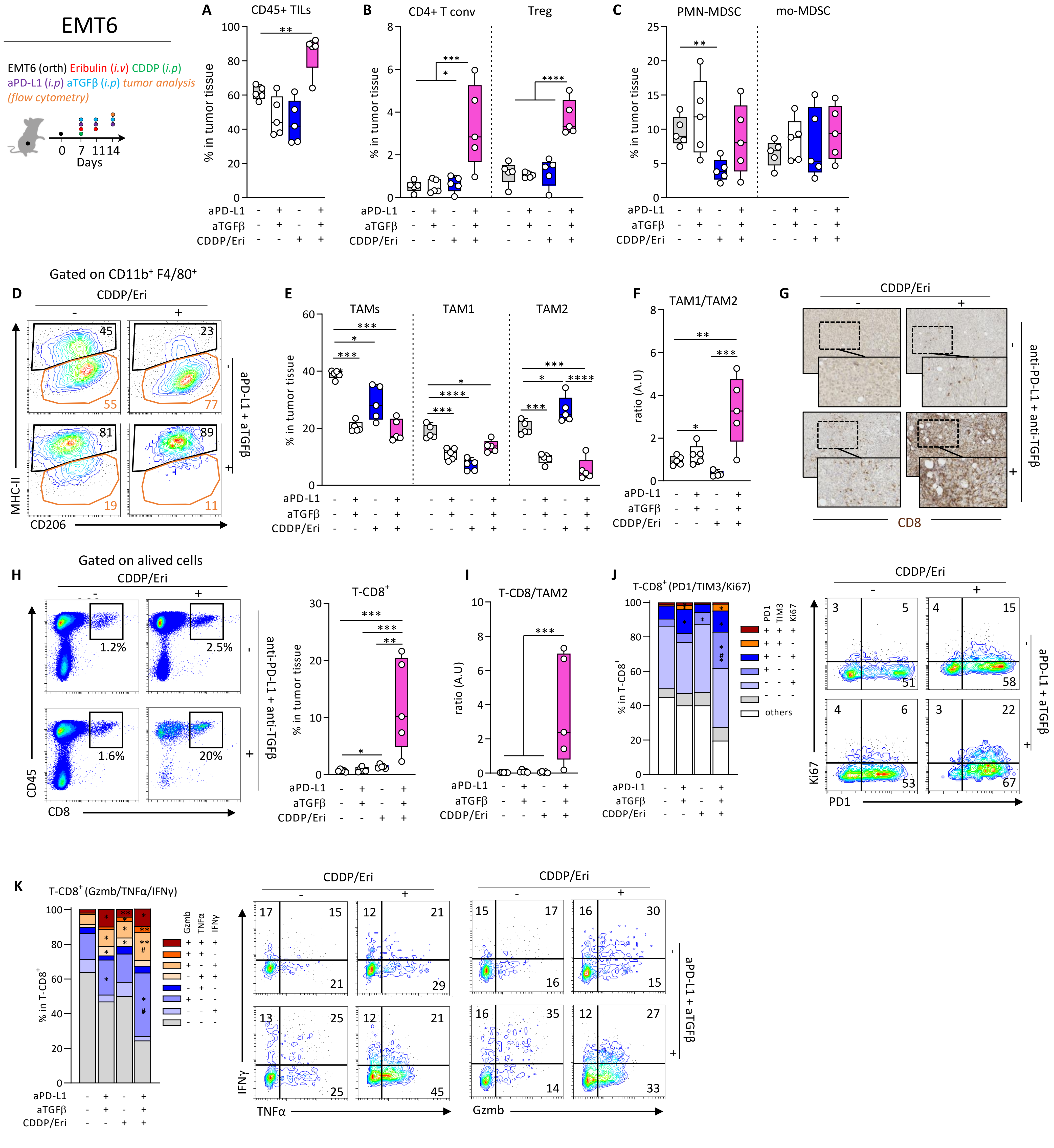

Supplementary Figure 7

Supplementary Figure 8:

A. Kaplan-Meier curves of overall survival (OS) according to expression of TGFB1 metagene. Green curve: patients with TGFB1 metagene low tumors, red curve: patients with TGFB1 metagene high tumors. Ticks denote censored data

B. Kaplan-Meier curves of overall survival (OS) according to expression of immune signature CYTOX. Green curve: patients with CYTOX low tumors, red curve: patients with CYTOX high tumors. Ticks denote censored data.

C .Kaplan-Meier curves of overall survival (OS) according to expression of immune signature GEP. Green curve: patients with GEP low tumors, red curve: patients with GEP high tumors. Ticks denote censored data.

D. Heatmap showing correlations between TGB1 gene, TGFB1 metagene and different immune signatures (CXCL10, IDO1, STAT1, CXCL9, TILS, HLA.DRB1, IFNG) for each subtype of Burstein classification of triple negative breast cancer (TNBC) : Basal-Like Immune-Activated (BLIA), Basal-Like Immune-Suppressed (BLIS), Luminal-AR (LAR), Mesenchymal (MES).

E. Barplots showing repartition of patients into Basal-Like Immune-Activated (BLIA), Basal-Like Immune-Suppressed (BLIS), Luminal-AR (LAR), Mesenchymal (MES) subtypes for the combination of GEP signatures and TGFB1 metagene (Low/High, High/Low, High/High and Low/Low). Pie charts showing repartition (expressed in percentage) of patients into Basal-Like Immune-Activated (BLIA), Basal-Like Immune-Suppressed (BLIS), Luminal-AR (LAR), Mesenchymal (MES) subtypes for the combination of GEP signatures and TGFB1 metagene (first line : Low/High, High/Low, High/High and Low/Low).

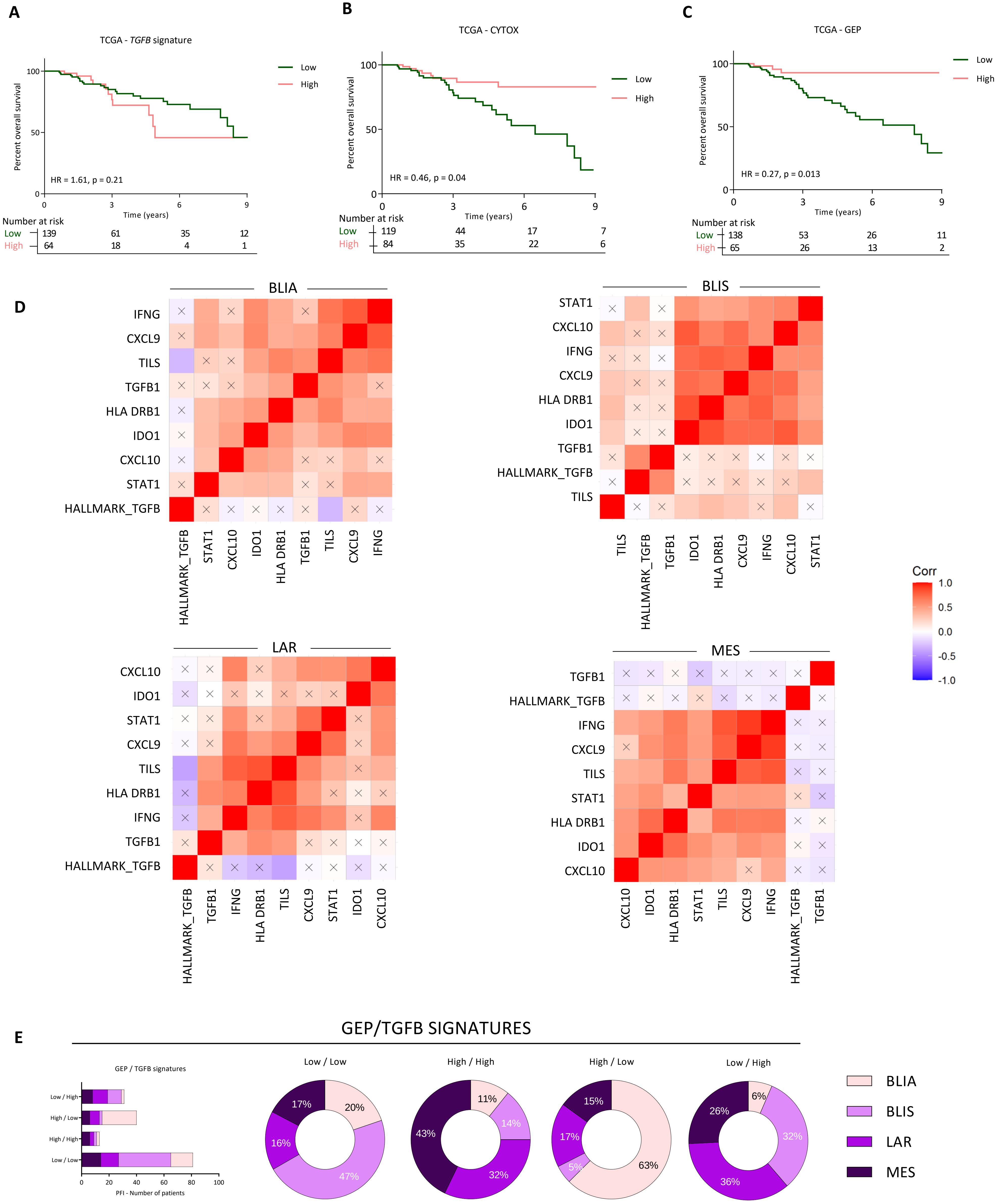

Supplementary Figure 8

Supplementary Figure 9:

A-C.MFM-223, HCC38, MDA-MB-231, MDA-MB-468, HCC1937, BT-549, and DU4475 human cell lines were treated with various doses of Eri and CDDP. Forty-eight hours after treatment, the half-maximal inhibitory concentration (IC50) was determined by analysis of cell viability by MTT for Eri (**A**) and CDDP (**B**). The IC50s of different cell lines are presented (**C**).

D. Cells were treated with various doses of cisplatin and Eri for 48 h. Viability was assessed by an MTT assay. The heatmap represents the MTT results, showing the mean percentage of adherent cells for each condition from three independent experiments.

E. MFM-223, HCC38, MDA-MB-231, MDA-MB-468, HCC1937, BT-549, and DU4475 human cell lines were seeded. After forty-eight hours, *TGFB1* expression was evaluated by RT-qPCR (at least 2 independent experiments, n = 3/experiments, one-way ANOVA test). *$p < 0.05$, **$p < 0.01$, ***$p < 0.001$, and ****$p < 0.0001$.

Supplemental methods:

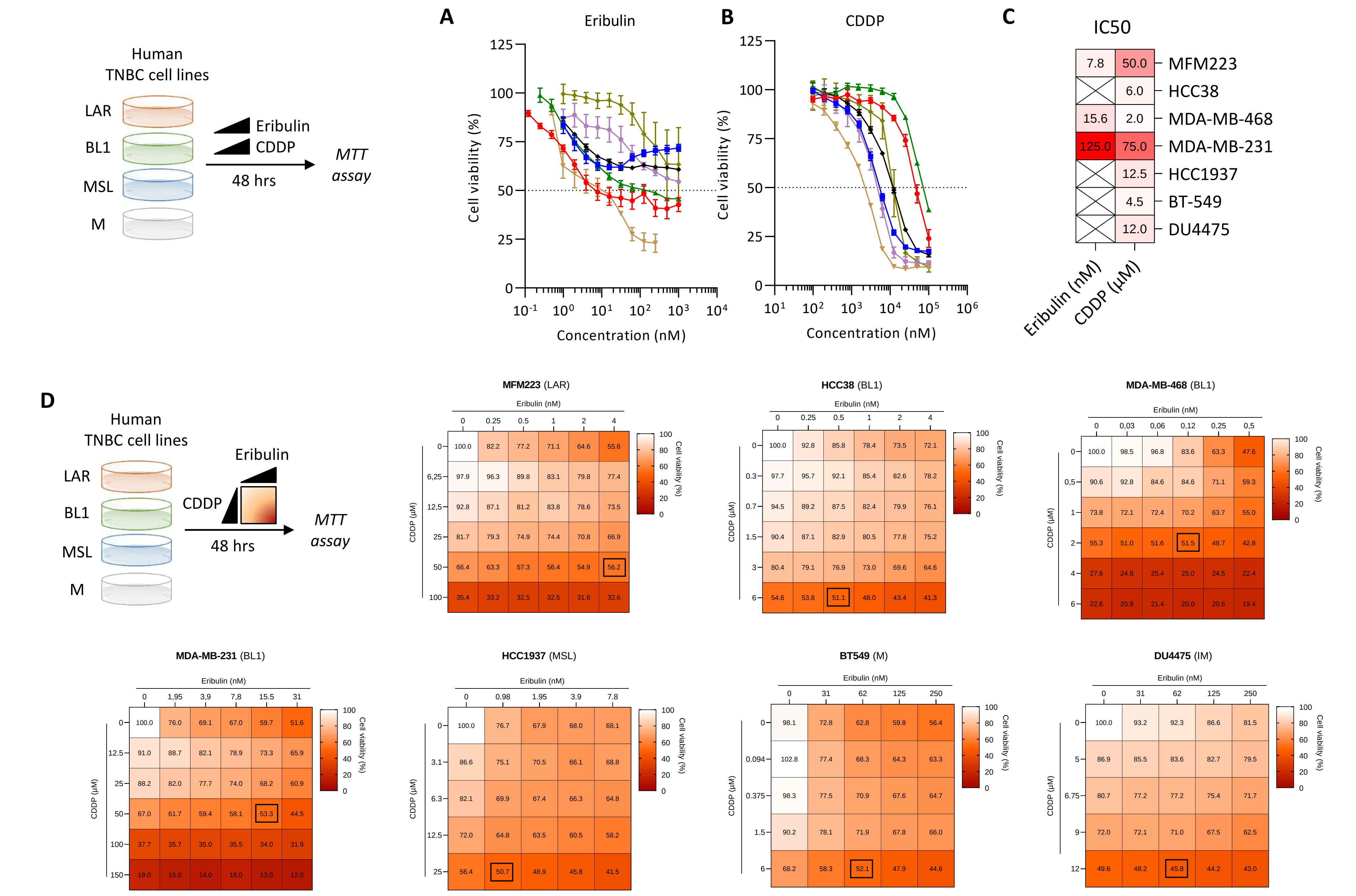

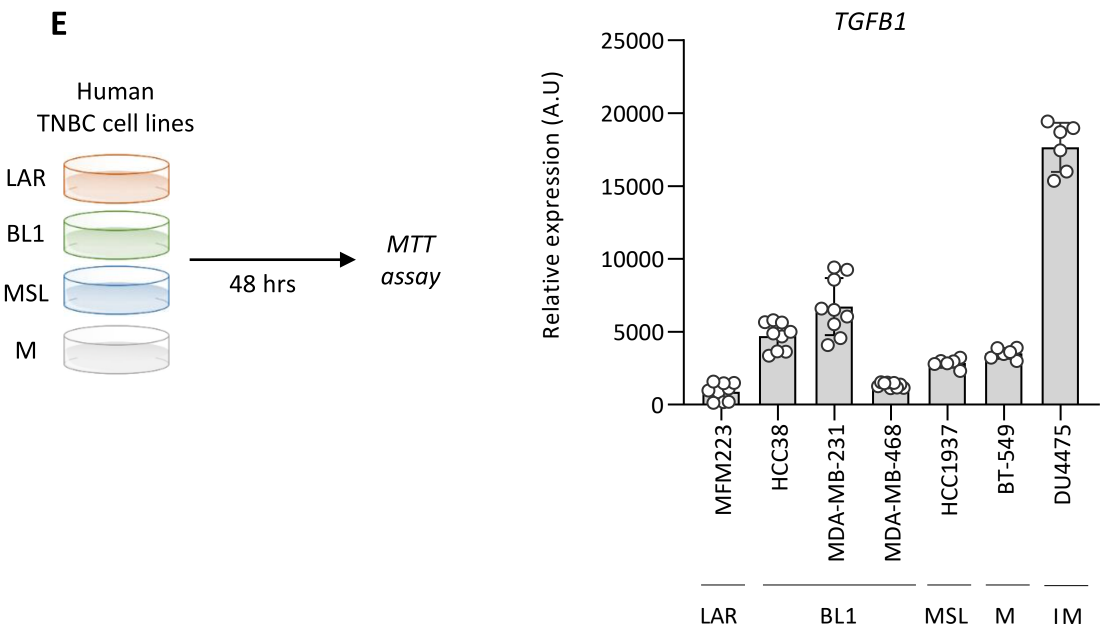

	MFM223	HCC38	MDA-MB-231	MDA-MB-468	HCC1937	BT-549	DU4475
MFM223		****	****	ns	***	****	****
HCC38			**	****	ns	ns	****
MDA-MB-231				****	****	****	****
MDA-MB-468					**	***	****
HCC1937						ns	****
BT-549							****
DU4475							

Supplementary Figure 9

Supplementary Material and Methods:

Viability assays

Murine cell lines were seeded in 96-well plates at 10,000 cells/well (4T1) and 5,000 cells/well (EMT6) the day before treatment with chemotherapies. Human cell lines were seeded in 96-well plates at 20,000 cells/well (MDA-MB-231 and MDA-MB-468), 6,500 cells/well (HCC38), 10,000 cells/well (HCC1937), 12,500 cells/well (BT-549 and MFM-223), and 25,000 cells/well (DU4475). After treatment, cells were washed twice with PBS and fixed with 100% ethanol for 30 min before crystal violet (Sigma-Aldrich) staining. Cells were then resuspended in a 50% ethanol/10% SDS solution. The optical density (OD) was read at 575 nm with a Spark 10M spectrophotometer (Tecan Life Sciences). For MTT assays, cells were incubated for 3 hours at room temperature with MTT, according to the manufacturer's protocol. Cells were then incubated over night at 37°C in a SDS/HCl solution, and OD was read at 570 nm with a Spark 10M spectrophotometer (Tecan Life Sciences).

Cell death determination

For Annexin-V/DAPI labeling, cells were seeded in 24-well dishes at 54,000 cells/well (4T1) and 28,000 cells/well (EMT6). After treatment with chemotherapies for 24 or 48 h, cells were harvested and washed twice with Annexin-V buffer and then stained using the Annexin-V (FITC)/7-AAD kit (Miltenyi Biotech), according to the manufacturer's protocol. Annexin-V binding and DAPI incorporation were detected using a Cytoflex cytometer (Beckman Coulter) and analyzed using CytExpert software (Beckman Coulter). The proportion of dead cells was evaluated by counting Annexin-V$^+$/DAPI$^+$ cells as necrotic, and Annexin-V$^+$/DAPI$^-$ cells as apoptotic. Annexin-V$^-$/DAPI$^-$ cells were considered alive.

Detection of immunogenic cell death (ICD), MHC-I, PD-L1 and stress-marker induction

ICD analysis was performed in parallel with cell death analysis. The analysis of cell surface calreticulin (CRT), H2Kd/H2Dd, and PD-L1 was performed by flow cytometry. For CRT staining, cells were incubated with primary mouse anti-CRT for 30 min at 4°C. Cells were washed twice with Flow Staining Buffer, and the secondary antibody, Alexa Fluor 647-labeled goat-anti-mouse IgG (H+L) (A21036, Life Technologies), was added, and the cells were incubated for 15 min at 4°C. Cells were washed twice with Flow Staining Buffer (eBioscience) and stained with a DAPI solution (Miltenyi). The proportion of DAPI$^-$ cells with surface CRT expression was detected using a Cytoflex cytometer (Beckman Coulter) and analyzed using CytExpert software (Beckman Coulter Inc., Brea, CA, USA). CRT positivity was evaluated using an isotype control antibody (ab170190, Abcam). For H2kd/H2Dd and PD-L1 staining,

cells were incubated with FITC anti-mouse H-2Kd/H-2Dd antibody (Biolegend) or PE anti-mouse CD274 (B7-H1, PD-L1) antibody (Biolegend) for 15 min at room temperature. Cells were washed twice with Flow Staining Buffer (eBioscience) and stained with a DAPI solution (Miltenyi). The proportion of H2Kd/H2Dd or PD-L1 positive cells was detected using a Cytoflex cytometer (Beckman Coulter) and analyzed using CytExpert software. HMGB1 and CXCL10 levels in culture supernatants were measured by ELISA, according to the manufacturer's instructions (Chondrex and RD systems). ATP levels in culture supernatants were measured by luminescence (CellTiter-Glo® Luminescent Cell Viability Assay, Promega), according to the manufacturer's instructions.

MLEC reporter cells

TGFβ levels in the culture supernatant were assayed using Mouse Lung Epithelial Cells (MLEC) reporter cells. These cells were stably transfected with an expression construct containing a truncated plasminogen activator inhibitor-1 (PAI-1) promoter fused to the firefly luciferase reporter gene[18]. MLEC cells were seeded in a 96-well plate at 1.6×10^3 cells per well. After 5 hours, the supernatant from the 4T1-treated CDDP (4 µM) or TGFβ (10 ng/mL) for 48 hours was added to the reporter cells. In parallel, MLEC cells were treated with a dose effect of TGFβ recombinant to create a standard range. After 20 hours at 37°C, lysis buffer was added to the cells for 15 minutes at RT, and then 100 µL of the lysate was analyzed on a white plate by luminescence reading with a Spark 10M spectrophotometer (Tecan Life Sciences). The TGFβ concentration was calculated using the standard curve.

CRISPR-Cas9 Transfection

Two crRNAs targeting Tgfb1 were designed and purchased from Integrated DNA Technologies: AB (5'-TGCGGTCCACCATTAGCACG-3') and AD (5'-CACCTGCAAGACCATCGACA-3'). Ribonucleoprotein (RNP) complexes were assembled according to the manufacturer's protocol (Alt-R CRISPR-Cas9 System, Integrated DNA Technologies). 4T1 cells were seeded at a density of 250,000 cells/mL in antibiotic-free culture medium and were transfected with the RNP complexes using the Cas9 Lipofectamine CRISPRMAX kit (Fisher Scientific). Following 48 hours of transfection, cells were individually sorted, and clones (R1 and R2) were subcultured for expansion. Knockout of *Tgfb1* in each subclone was confirmed by DNA sequencing and protein secretion analysis using the LegendPlex assay (BioLegend).

Platinum dosage

Analysis of platinum (Pt) was performed using an inductively coupled plasma spectrometer (Thermo Scientific©) with high-resolution mass detection (Element 2). All solutions were prepared in HNO_3 2%. Nitric acid (67%, Optima grade) was obtained from Fisher Scientific©. Calibration standard solutions were prepared from a certified solution with a platinum concentration of 1000 mg/L (TechLab©). A precisely weighed amount of each tissue was mineralized with 2 mL of concentrated HNO_3 using a microwave (Ultrawave, Milestone©). After mineralization, the volume is filled to 5 mL with ultra-pure water. After analysis, the resulting concentrations of platinum enabled calculation of the platinum concentration per kg of tissue for each sample.

TGFβ1 level assay

Murine 4T1 WT and 4T1 *Tgfb1*$^{-/-}$ cells were seeded in 96-well plates at 10 000 cells/well for 48 hours. The supernatants were removed from the cells and concentration of the cytokine TGFβ1 was analyzed according to the manufacturer's instructions for the bead-based immunoassay (LegendPlex, Biolegend). Test samples were incubated with beads at room temperature for 2 hours with agitation. After washing, biotin-coupled detection antibodies specific to a given cytokine was added for 1 hour then followed by the addition of a streptavidin-PE solution for 30 minutes. The plate was washed and finally resuspended in an assay buffer for reading on the Cytoflex instrument (Beckman Coulter). The final concentration of analyte was calculated using the LegendPlex software.

Western blotting

Whole-cell lysates were prepared by lysing cells with RIPA buffer (Sigma) in the presence of complete protease inhibitors (Roche). Proteins were extracted by 3 x 10-minute incubations in ice, interspersed with vortexing, followed by 20-minute centrifugation at 20000 g at 4°C. After the Lowry assay, proteins were deposited in the gel at the same quantity (10µg) in the different conditions and were separated by SDS-polyacrylamide gel electrophoresis and electroblotted onto a nitrocellulose membrane (GE Healthcare). After incubation for 1 hour at RT in 5% w/v nonfat milk in Tris-buffered saline-0.1% Tween-20 (TBST), membranes were incubated overnight at 4°C with the primary antibody diluted in TBST with BSA (5% w/v) (TBST-BSA). Membranes were washed three times in TBST and then incubated with the secondary antibody for 1 hour at RT in TBST-BSA. Membranes were washed again three times before analysis using a chemiluminescence detection kit (Santa Cruz Biotechnology, Thermo Fisher Scientific). HSC 70 was used as a loading control protein. The primary antibodies used are available in Supplementary Table 1.

Macrophage differentiation

Human blood was obtained from the French Blood Transfusion Service in Dijon. Peripheral Blood Mononuclear Cells (PBMC) were obtained from buffy coat by gradient centrifugation using Ficoll. The monocytes were isolated from PBMC using human CD14 microbeads (Miltenyi Biotech) according to the manufacturer's instructions. Monocytes were seeded in 24-well plates at 250,000 cells per well, and the supernatant was discarded two hours later. Monocytes were incubated for 6 days in AIMV medium supplemented with CSF-1 (100 ng/mL) to obtain macrophages.

Conditioned medium preparation

MDA-MB-468 were seeded in a T25 flask at $1.5x10^6$ cells and treated after 24 hours with CDDP (4µM) and Eri (0.5nM) for 48 h. The media were harvested and filtered through an 0.22µm filter. The CCD-Lu19 fibroblastic cell line was seeded in 24-well plates at 15000 cells/well. After 24 hours, differentiated macrophages and fibroblasts were treated with galunisertib (100 nM) for 2 hours, and then the prepared conditioned medium was added. After 48 hours, fibroblasts and macrophages were recovered in Trizol for RNA extraction.

MMTV-PyMT Mice

Mice were bred and maintained in the animal facility of the University of Burgundy according to the center instructions; all the experiments were carried out following the instructions of the FELASA. Experimental designs involving animals were approved by the Ethics Committee of the University of Burgundy (projects #17461, #14557, and #22358). B6.FVB-Tg (MMTV-PyVT)634Mul/LellJ (MMTV-PyMT) mice were obtained from the Jackson Laboratory. Mouse genotyping was performed using extraction solution (E7526; Sigma-Aldrich) and neutralization solution B (N3910; Sigma-Aldrich) and with PCR primer sets for the detection of the MMTV-PyMT transgene.

Female mice were monitored by palpation, and tumor growth was measured every 2 days. Tumor surface (mm^3) was calculated with the following equation:

$$\frac{small\ axis^2 \times major\ axis}{2}$$

When a tumor reached a minimum size of 50 mm^3, mice were randomly assigned to a treatment group, and the treatment was identical to that of BALB/c mice. The so-called "target tumor" was measured twice a week, and the appearance of new tumors was also analyzed and measured.

Histology and immunohistochemistry

Tumors were collected 3 or 8 days after randomization/treatment, fixed in PFA, and embedded in paraffin by the pathology laboratory. Four-micron slices were cut from formalin-fixed paraffin-embedded tumor samples. The tissues embedded in paraffin were cut on an AutoSection™ automated microtome. 4 µm thick sections were deposited on a glass slide tomo, deparaffinized, and stained with a Leica AutoStainer Link 48. For CD8a, alpha smooth muscle (αSMA), and CD31 staining, slides with a thickness of 4µm were deparaffinized and stained using a Dako PT link (Agilent). Briefly, slides were deparaffinized using a pH9 buffer for 20 minutes at 97°C. After cooling, slides were washed in wash buffer (Agilent) twice for 5 minutes. Peroxydase blocking was performed by incubating samples in the S2023 solution (Agilent) for 5 minutes. Blocking was performed with normal goat serum (MP-7451-50 kit, Vector Laboratories) for 20 minutes. Then anti-murine CD8 (1/200, polyclonal HS-361 00, Sysy Ab), anti-murine αSMA (1/500, D4K9N, Ozyme), and anti-murine CD31 (1/2500, EPR17259, Abcam) antibodies were added for 30 minutes at RT. ImmPRESS HRP goat polymers (MP-7451-50 kit, Vector Laboratories) were added for 15 minutes at RT after two washing steps. DAB (SM803) was then added to the samples for 5 minutes. After two new washing steps, slides were finally incubated with hematoxylin (Enzo) for 5 minutes and permanently mounted manually.

For collagen staining with the Masson-Goldner Trichrome kit, slides with a thickness of 4µm were deparaffinized and stained using a PT link (Agilent). Briefly, slides were deparaffinized using a pH9 buffer for 50 minutes at 95°C. After cooling, slides were washed in wash buffer (Agilent) twice for 5 minutes. The blades were covered with ferric hematoxylin, according to Weigert, for 10 minutes. The slides were washed with water for 5 minutes, then covered with picric acid for 5 minutes. The slides were washed with distilled water for 30 seconds, then covered with ponceau fuchsin according to Masson for 10 minutes. Phosphomolybdic acid was added for 4 minutes, and a light green solution was added for 2 minutes. The slides were dried and then permanently mounted using a Leica automated coverslipper. The slides were scanned by an Olympus Slideview VS200 at 20X magnification. The analysis of the scans was performed by a pathologist by zoning blinded samples. The scans were analyzed with the Qupath software, using a script available on request.

Tumor cells, CD45^{+} TIL and CAF isolation

Tumors were collected 4 days after randomization/treatment. After dissection, tumors were mechanically and enzymatically dissociated using a mouse tumor dissociation kit, according to the manufacturer's recommendations (Miltenyi Biotech). Tumor cells and CD45+ TILs were isolated by using, respectively, the Tumor Cell Isolation Kit and CD45 (TIL) MicroBeads (Miltenyi Biotech) according to the manufacturer's instructions. After CD45+ TIL isolation, unlabeled cells were collected, and

fibroblast-associated cancer was isolated by using the Tumor-Associated Fibroblast Isolation Kit (Miltenyi Biotech) according to the manufacturer's instructions. The details of this method are available in our published protocol[19]. $CD45^+$ TIL fraction was stained in Flow Cytometry Staining Buffer (FSB, eBioscience) with specific antibodies according to the manufacturer's recommendations, for 15 min at RT in the dark, washed twice in FSB. Then, among CD45+ cells, TAM1, TAM2, PMN-MDSC, and Mo-MDSC populations were sorted by flow cytometry (Cell Sorter Aria III, BD).

Public datasets

Metabric dataset consisted in the discovery data set of this cohort (n = 997 patients), generated by the Molecular Taxonomy of Breast Cancer International Consortium. Normalized microarray data, obtained with the Illumina HT 12 platform, were requested and downloaded from the European Genome-phenome Archive (EGA) under the identifier EGAD00010000210. Overall Survival (OS) and clinical information were also available.

As to BRCA TCGA dataset (n=1093), RNAseqV2 data with RSEM normalization and clinical data were downloaded from the TCGA data portal (https://portal.gdc.cancer.gov/).

Six transcriptomic signatures were computed as a metagene by taking the mean expression of corresponding genes. Three signatures are related to IFN pathways or checkpoint inhibitors: IFNγ gene expression, extended immune gene signature (EIG) and T cell-inflamed gene expression profile (GEP) and four are related to T cell immune infiltrate: cytotoxicity (CYTOX), Th1 orientation (Th1), cytotoxic lymphocytes (CTL) and CD3e expression. TILS signature was computed as described elsewhere [20]. Briefly, this score was generated using four 10-gene signatures, each signature representing respectively lymphoid, myeloid, stromal and tumor cells. Each signature was quantified through a score based on 10 genes selected as characteristics of the corresponding cellular subtype. Then, the TILS score was defined as the ratio of the sum of lymphoid and myeloid scores to the total of the 4 cell-type scores.

TGFβ signature was estimated through a ssGSEA (single-sample Gene Set Enrichment Analysis, GSEA) aalysis based on "HALLMARK_TGF_BETA_SIGNALING" pathway provided by MSigDB database (https://www.gsea-msigdb.org/gsea/msigdb/cards/HALLMARK_TGF_BETA_SIGNALING.html). ssGSEA analysis was performed using GSVA R package [21].

Supplementary Table 1

REAGENT or RESOURCE SOURCE IDENTIFIER		
**Antibodies (clone)**	**Source**	**Identifier**
Anti-mouse CD8a (2.43)	Bioxcell	Cat#BE0061
Anti-mouse TGF-β (1D11.16.8)	Bioxcell	Cat#BE0057
Anti-mouse CD274 (10F.9G2)	Bioxcell	Cat#BE0101
Rat IgG2a, k isotype control (2A3)	Bioxcell	Cat#BE0089
Rat IgG2b, k isotype control (LTF-2)	Bioxcell	Cat#BE0090
Anti-mouse PD-1 VioBlue (HA2-7B1)	Milteny	Cat#130-121-437
Anti-mouse Ly6C VioGreen (1G7.G10)	Miltenyi	Cat#130-102-207
Anti-mouse F4/80 BV605 (BM8)	Biolegend	Cat#123133
Anti-mouse Ly6G BV650 (1A8)	Biolegend	Cat#127641
Anti-mouse CD11b BV785 (M1/70)	Biolegend	Cat#101243
Anti-mouse HLA-DR Alexa fluor 488 (M5/144.M5.2)	Biolegend	Cat#107615
Anti-mouse PD-L1 PE (10F.9G2)	Biolegend	Cat#124308
Anti-mouse PD-L1 (2096C)	RD system	Cat#MAB90781
Anti-mouse CD45 APC (REA737)	Miltenyi	Cat#130-110-660
Anti-mouse CD45 Alexa Fluor 647 (30F11)	Biolegend	Cat#103124
Anti-mouse CD4 VioGreen (REA604)	Miltenyi	Cat#130-118-693
Anti-mouse CD8 BV605 (J3-6-7)	Biolegend	Cat#100744
Anti-mouse Tim3 BV785 (RMT3-23)	Biolegend	Cat#119725
Anti-mouse Foxp3 VioBright 515 (REA788)	Miltenyi	Cat#130-111-681
Anti-mouse Ki67 PE (REA183)	Miltenyi	Cat#130-120-557
Anti-mouse NKp46 PE-Vio770 (REA815)	Miltenyi	Cat#130-112-203
Anti-mouse CD3 Alexa Fluor 700 (17A2)	Biolegend	Cat#100216
Anti-mouse TNFa BioBright 515 (REA636)	Miltenyi	Cat#130-109-718
Anti-mouse Granzyme B PE (REA226)	Miltenyi	Cat#130-116-486
Anti-mouse IFNg PE-Cy7 (XMG1.2)	Biolegend	Cat#505826
Anti-mouse CD45 VioBlue (REA737)	Miltenyi	Cat#130-110-664
Anti-mouse CD140a BV605 (APA5)	Biolegend	Cat#135916
Anti-mouse CD140b APC-Vio770 (REA634)	Miltenyi	Cat#130-118-469
Anti-mouse CD31 BV785 (390)	Biolegend	Cat#102435
Anti-mouse FAPa Alexa Fluor 488 (polyclonal)	Bioss antibodies	Cat#bs-5758R-A488
Anti-mouse CD26 PE (REA1196)	Miltenyi	Cat#130-122-775
Anti-mouse CD49f PE-Vio770 (REA518)	Miltenyi	Cat#130-123-549
Anti-mouse CD24 PE-Vio770 (REA743)	Miltenyi	Cat#130-110-690
Anti-mouse CD326 (EpCAM) PE-Vio770 (REA977)	Miltenyi	Cat#130-117-755
Anti-mouse podoplanin APC (8.1.1)	Biolegend	Cat#127410
Anti-H2kd-H2Dd FITC (34-1-2S)	Biolegend	Cat#114706
Anti-EIF2a (D7D3)	Cell Signaling	Cat#5324
Anti-phospho Ser 51 EIF2a (polyclonal)	Cell Signaling	Cat#9721
Anti-beta actin (AC-15)	Sigma Aldrich	Cat#A1978
Anti-Calreticulin (FMC75)	Abcam	Cat#ab22683

Anti-phospho Ser172 TBK1 (D52C2)	Cell Signaling	Cat#5483
Anti-TBK1 (Polyclonal)	Cell Signaling	Cat#3013
Anti-smad2/3 (D7G7)	Cell Signaling	Cat#8685
Anti-phospho Ser465/467 smad2/3 (D27F4)	Cell Signaling	Cat#8828
Anti-LC3I/II (D3UC4)	Cell Signaling	Cat#12741
Anti-Biotin (polyclonal)	Jackson Immuno Research	Cat#711-065-152
Anti-Rabbit Alexa Fluor 488 (polyclonal)	Invitrogen	Cat#A32731
Anti-Rabbit Alexa Fluor 568 (polyclonal)	Invitrogen	Cat#A-11011
Anti-Mouse Alexa Fluor 488 (polyclonal)	Invitrogen	Cat#A-11029
Anti-Rabbit Alexa Fluor 594 (polyclonal)	Jackson Immuno Research	Cat#111-585-144
Anti-HSC70 (B-6)	Santa Cruz Biotechnology	sc-7298
Peroxidase-conjugated AffiniPure Goat Anti-Mouse IgG (H+L)	Jackson Immuno Research	115-035-003
Peroxidase-conjugated AffiniPure Goat Anti-Rabbit IgG (H+L)	Jackson Immuno Research	111-035-144
Anti-CD8a (polyclonal)	Sysy	HS-361 003
anti CD31 (EPR17259)	Abcam	ab182981
Anti-alpha-Smooth Muscle Actin (D4K9N)	Cell Signaling	Cat#19245
**Chemicals, Peptides, and Recombinant Proteins**		
Eribulin		
Cisplatin		
Doxorubicin		
Galunisertib	Selleckchem	Cat#S2230
DAPI	Miltenyi	Cat#130-111-570
Fixable Viability Dye eFluor™ 780	Thermo Fisher	Cat# 65-0865-14
Viobility 405/452 Fixable Dye	Miltenyi	Cat#130-109-816
Ethidium bromide solution	Sigma aldrich	Cat#E1385
PMA	Sigma Aldrich	Cat#P1585
Ionomycin	Sigma Aldrich	Cat#I9657
Brefeldin A	Biolegend	Cat#420601
Recombinant Mouse TGF-beta 1 Protein	Biotechne	7666-MB
Recombinant Human TGF-beta 1 Protein	Biotechne	240-B
IFNb1 mouse recombinant	Biolegend	Cat#581302
Recombinant Human IFNb Protein	R&D	8499-IF-010
ATP solution	Sigma aldrich	Cat#A6559
M-MLV REVERSE TRANSCRIPTASE	Fisher Scientific	Cat#10338842
DATP, DCTP, DGTP, DTTP nucleotides	Promega	Cat#U1420
Rnase OUT	Fisher Scientific	10154652
cGAMP	Invivogen	vac-nacga23
RIPA Buffer	Sigma aldrich	R0278-50ML
Western Blotting Luminol Reagent	Santa Cruz Biotechnology	sc-2048

SuperSignal West Femto Maximum Sensitivity Substrate	Thermo Fisher	34096
SYBR Master Mix PCR Power SYBR Green	Fisher Scientific	10219284
Ambion Trizol Reagent 100ml	Fisher Scientific	12044977
Mycoalert Detection Kit 50 test	Lonza	LT07-418
Mycoalert Assay Control Set	Lonza	LT07-518
Complete Protease Inhibitor Cocktail	Sigma aldrich	11697498001
**Critical Commercial Assays**		
Tumor Dissociation Kit, mouse	Miltenyi Biotec	Cat#130-096-730
CD45 (TIL) MicroBeads, mouse	Miltenyi Biotec	Cat#130-110-618
Tumor Cell Isolation Kit	Miltenyi Biotec	Cat#130-110-187
Tumor-Associated Fibroblast Isolation Kit	Miltenyi Biotec	Cat#130-116-474
Dead Cell Removal Kit	Miltenyi Biotec	Cat#130-090-101
CELLTITER-GLO® LUMINESCENT CELL VIABILITY ASSAY	Promega	Cat#G7571
HMGB1 Detection Kit	Chondrex	Cat#6010
Mouse CXCL10/IP-10/CRG-2 DuoSet ELISA	RD system	Cat#DY466-05
LEGENDplex™ Mouse TGF-β1	Biolegend	Cat#740692
Vybrant MTT Cell proliferation Assay Kit	Thermo Fisher	Cat#V13154
AV/7AAD	Miltenyi	Cat#130-092-052
Crystal violet solution	Sigma aldrich	Cat#V5265
FoxP3 Staining Buffer Set	Miltenyi	Cat#130-093-142
Masson-Goldner Trichrome Staining Kit	DiaPath	Cat#010224
**Experimental Models: Cell Lines**		
4T1	ATCC	Cat#ATCC® CRL-2539™
TS/A	Millipore	Cat#SCC177
MC38		
EMT6	ATCC	Cat#ATCC® CRL-2755™
MLEC		
MDA-MB-231	ATCC	Cat#ATCC® CRM-HTB-26™
MDA-MB-468	ATCC	Cat#ATCC® HTB-132™
MFM-223	Sigma Aldrich	Cat#98050130-1VL
HCC38	ATCC	Cat#ATCC® CRL-2314™
HCC1937	ATCC	Cat#ATCC® CRL-2336™
DU4475	ATCC	Cat#ATCC® HTB-123™
BT-549	ATCC	Cat#ATCC® HTB-122™
CCD-19Lu	ATCC	Cat#ATCC® CCL-210™
**Experimental Models: Organisms/Strains**		
BALB/c mice	Charles River	CRL:BALB/cAnNCrl
FVB mice	Charles River	FVB/NCrl
Nude NMRI mice	Charles River	Crl:NMRI-Foxn1nu
**Software and Algorithms**		
GraphPad Prism version 7.00	GraphPad Software	https://www.graphpad.com/scientific-software/prism/
CytExpert softwar	Beckman Coulter	https://www.beckman.fr/flow-cytometry/instruments/cytoflex/software

Fiji (ImageJ)		https://fiji.sc/
Applied Biosystems QuantStudio 6/7 Pro Real-Time PCR Systems	Applied Biosystems	https://www.thermofisher.com/fr/fr/home/global/forms/life-science/quantstudio-6-7-pro-software.html
ImageLab	Biorad	https://www.bio-rad.com/fr-fr/product/image-lab-software?ID=KRE6P5E8Z

Supplementary Table 2

RT-qPCR primers

Genes	Species	Forward	Reverse
Actinb	Mus musculus	ATGGAGGGGAATACAGCCC	TCTTTGCAGCTCCTTCGTT
Atf4	Mus musculus	CATGGGGCCTTTAGGACGAT	GCCCTAAACCCGCCCTTTAT
Cd274	Mus musculus	GCTTCTCAATGTGACCAGCA	GAGGAGGACCGTGGACACTA
Cd3e	Mus musculus	AAGTAATGAGCTGGCTGCGT	ATGTTCTCGGCATCGTCCTG
Cd8a	Mus musculus	TTCTGTCGTGCCAGTCCTTC	GCTTTCGGCTCCTGTGGTAG
Cxcl10	Mus musculus	CCAAGTGCTGCCGTCATTTT	TTCATCGTGGCAATGATCTCAAC
Cxcl9	Mus musculus	TGCCATGAAGTCCGCTGTTC	CTAGGGTTCCTCGAACTCCAC
Gzmb	Mus musculus	CATGCTGCTAAAGCTGAAGAGT	GCACGTTTGGTCTTTGGGTC
H2db	Mus musculus	AGTGGTGCTGCAGAGCATTACAA	GGTGACTTCACCTTTAGATCTGGG
H2kb	Mus musculus	GCTGGTGAAGCAGAGAGACTCAG	GGTGACTTTATCTTCAGGTCTGCT
Ifng	Mus musculus	TGAGCTCATTGAATGCTTGG	ACAGCAAGGCGAAAAAGGAT
Irf1	Mus musculus	AATTCCAACCAAATCCCAGG	AGGCATCCTTGTTGATGTCC
Tap1	Mus musculus	CTGGGAAAAGTGCATCAGTCAC	AATGAGACAAGGTTGCCGCTG
Tapasin	Mus musculus	AACCTGCCACACTGCTACTG	AGACTTCTGGCCCACTTCG
Acta2	Mus musculus	CCAGCCATCTTTCATTGGGATG	TACCCCCTGACAGGACGTTG
Tgfbr2	Mus musculus	ACGTTCCCAAGTCGGATGTG	GTTTCAGTGGATGGATGGTCCT
Tgfbr3	Mus musculus	CTGCCAAGGGAGGTTCACAT	AGAACCCTCCGAAACCAGGA
Tgfb1	Mus musculus	ACTGGAGTTGTACGGCAGTG	GGGGCTGATCCCGTTGATTT
Serpin1	Mus musculus	GTTCATCGCTGCACCCTTTG	CTGCTCTTGGTCGGAAAGACT
Lrrc15	Mus musculus	TCCTTCTTATTGACCCGGCAG	CGGGAACAGGTACATTCGCT
Tagln	Mus musculus	CCCAAATATGGAGCCTGTGTG	TGCCCATGGAAGTCTGCTTG
Col8a1	Mus musculus	TGGACCCAAAGGGGAAATCG	CACCTTTTGCTCCTGGTTGC
Crabp1	Mus musculus	CGCTACCCTAGCGACTCAAG	AGCTCTCGGGTCCAGTAAGT
Sdc1	Mus musculus	CCAGAGGAGACAGAGCCTAAC	CTGATTGCTCTGCGCTTTGC
Cav1	Mus musculus	AATCACAGCCCAGGGAAACC	TGAAGCTGGCCTTCCAGATG

Supplementary Table 2

Sfrp2	Mus musculus	AGGCCTTGGAAACCCATAGC	GGGACCCACAAGCTTAGACC
Cxcl12	Mus musculus	GGAGAAAGCTTTAAACAAGAGGCT	CTCCTGTAAGTTCCTCGGGC
Mmp3	Mus musculus	GTTCTGGGCTATACGAGGGC	TTCTTCACGGTTGCAGGGAG
Saa3	Mus musculus	AACTATGATGCTGCCCGGAG	GCTCCATGTCCCGTGAACTT
Tnxb	Mus musculus	ATGGCAGCTCAGTGCACCCCGTCTA	AAGACACCGTGGAGGCTGCAGAGGC
Ly6c1	Mus musculus	ACCCTTCTCTGAGGATGGACA	GATCCCTGATTGGCACACCA
cd55	Mus musculus	GAAAGACTGAGTTTTGCATCCCTCAAAAAAGAG	AAAACTGAGCAACTGGAGACCATACTAAATCC
Pi16	Mus musculus	GCCCATGAGAAACCCGGAAA	GGGACATCTTGGGGTACAGC
Pclaf	Mus musculus	TCCAATTGGCTTGTTGAGGG	AAGTAGAGTGCCAGGGAACG
Birc5	Mus musculus	TCGCCACCTTCAAGAACTGGCCCTTCCTGGA	GGCTTCTGACAATGCTTG
Stmn1	Mus musculus	TCGGTGTGCTGGGTTAATGG	TGGAAGAAGCCGCCACATAC
H2afz	Mus musculus	TGGTGGGCCGAACCG	ACGCATTTCCTGCCAACTCA
C1qtnf3	Mus musculus	GGCAGACAAATGGATGCAAAGT	TAGGCAAAAACCATCTAGCACCT
Aspn	Mus musculus	CTCTGACAAGGCCAGCATGAA	AGGGTTCACTGGCTCTTTCG
Mfap4	Mus musculus	TGAGCTGACACCATGAAGGC	CTGGGGCCATAGGGGTAGAT
Thbs4	Mus musculus	TGTGCGCTGTGTGAATTTGG	CATGGGTTCTGCTCTGGGTT
ACTINB	Homo sapiens	GTTGTCGACGACGAGCG	GCACAGAGCCTCGCCTT
CXCL10	Homo sapiens	GAGCCTACAGCAGAGGAACC	GCTGATGCAGGTACAGCGT
TGFB	Homo sapiens	CCGGTAGTGAACCCGTTGAT	GGAAATTGAGGGCTTTCGCC
SERPIN1	Homo sapiens	TGGTTCTGCCCAAGTTCTCC	CACCGTGCCACTCTCGTTC

CRISPR Cas9 sg

Mm.Cas9.TGFB1.1.AD	Mus musculus	CACCTGCAAGACCATCGACA
Mm.Cas9.TGFB1.1.AB	Mus musculus	TGCGGTCCACCATTAGCACG

PCR primers (used before Sanger Sequencing)

mTgfb1	Mus musculus	TGCTTTCTCCCTCAACCTCA	TAATTTCCTCCCGGTGACCC

Supplementary Table 3

**Target Identifier**	**Name**
NM_001111316.2	*Ptprc*
NM_026790.2	*Ifi27*
NM_027320	*Ifi35*
NM_133871.3	*Ifi44*
NM_008331.3	*Ifit1*
NM_008332.3	*Ifit2*
NM_010501.2	*Ifit3*
NM_177361.2	*Ifna1/5/6/12/13/14/15/16/b*
NM_010503.2	*Ifna2*
NM_010504.2	*Ifna4*
NM_010510.1	*Ifnb1*
NM_016849.4	*Irf3*
NM_016850.3	*Irf7*
NM_001159417.1	*Irf9*
NM_015783.3	*Isg15*
NM_010846.1	*Mx1*
NM_013606.1	*Mx2*
NM_026820.3	*Ifitm1*
NM_030694.1	*Ifitm2*
NM_025378.2	*Ifitm3*
NM_145211.2	*Oas1a*
NM_001347448.1	*Oas2*
NM_145226.2	*Oas3*
NM_013683.2	*Tap1*
NM_001025313.1	*Tapbp*
NM_010378.3	*H2-Aa*
NM_207105.3	*H2-Ab1*
NM_010382.2	*H2-Eb1*
NM_010398.3	*H2-T23*
NM_010380.3	*H2-D1*
NM_001001892.2	*H2-K1*
NM_010746.3	*Ncr1*
NM_001081445.1	*Ncam1*
NM_001099918.2	*Klrb1*
NM_001136068.2	*Klrc1*
NM_010654.4	*Klrd1*
NM_033078.4	*Klrk1*
NM_024253.4	*Nkg7*
NM_009018.1	*Raet1c*
NM_029975.2	*Ulbp1*
NM_010493.3	*Icam1*
NM_001359898.1	*Cd80*

NM_019388.3	*Cd86*
NM_021334.3	*Itgax*
NM_178706.5	*Siglech*
NM_011611.2	*Cd40*
NM_011616.2	*Cd40lg*
NM_001368415.1	*Cd47*
NM_008512.2	*Lrp1*
NM_001301811.1	*Irf8*
NM_008176.3	*Cxcl1*
NM_009140.2	*Cxcl2*
NM_203320.3	*Cxcl3*
NM_009141.3	*Cxcl5*
NM_023785.3	*Cxcl7*
NM_134156.3	*Cxcl8*
NM_008599.4	*Cxcl9*
NM_021274.2	*Cxcl10*
NM_018866.2	*Cxcl13*
NM_023158.7	*Cxcl16*
NM_011333.3	*Ccl2*
NM_013652.2	*Ccl4*
NM_013653.3	*Ccl5*
NM_011330.3	*Ccl11*
NM_011332.3	*Ccl17*
NM_011888.4	*Ccl19*
NM_016960.2	*Ccl20*
NM_009137.2	*Ccl22*
NM_019577.5	*Ccl24*
NM_020279.3	*Ccl28*
NM_009142.3	*Cx3cl1*
NM_011577.2	*Tgfb1*
NM_008871.2	*Serpin1*
NM_007392.3	*Acta2*
NM_010197.3	*Fgf1*
NM_008006.2	*Fgf2*
NM_007742.4	*Col1a1*
NM_007743.3	*Col1a2*
NM_009930.2	*Col1a3*
NM_007986.3	*Fap*
NM_008610.3	*Mmp2*
NM_013599.5	*Mmp9*
NM_010233.2	*Fn1*
NM_001025250.3	*Vegfa*
NM_009640.4	*Angpt1*
NM_007426.4	*Angpt2*
NM_008816.3	*Pecam1*

NM_053247.4	*Lyve1*
NM_011546.3	*Zeb1*
NM_015753.4	*Zeb2*
NM_011427.3	*Snai1*
NM_011658.2	*Twist1*
NM_011701.4	*Vim*
NM_009864.3	*Cdh1*
NM_001082960.1	*Itgam*
NM_010130.4	*Adgre1*
NM_008625.2	*Mrc1*
NM_007778.4	*Csf1*
NM_001037859.2	*Csf1r*
NM_010741.3	*Ly6C1*
NM_008396.3	*Itga2*
NM_009915.2	*Ccr2*
NM_009917.5	*Ccr5*
NM_008357.2	*Il15*
NM_001252382.1	*Irf5*
NM_001253872.1	*Itgal*
NM_001310438.1	*Ly6G*
NM_013650.2	*S100A8*
NM_001281852.1	*S100A9*
NM_001170395.1	*Cd163*
NM_008351.1	*Il12p35*
NM_031252.2	*Il23a*
NM_008353.2	*Il12rb1*
NM_032465.2	*Cd96*
NM_178687.2	*Cd226*
NM_001146325.1	*Tigit*
NM_008798.3	*Pdcd1*
NM_134250.2	*Havcr2*
NM_009843.4	*Ctla4*
NM_017480.2	*Icos*
NM_008479.2	*Lag3*
NM_009630.3	*Adora2a*
NM_027514.2	*pvr*
NM_021893.3	*Cd274*
NM_021396.2	*Pdcd1lg2*
NM_015790.3	*Icosl*
NM_007482.3	*Arg1*
NM_010927.4	*Nos2*
NM_009848.4	*Entpd1*
NM_011851.4	*Nt5e*
NM_008324.2	*Ido1*
NM_145949.2	*Ido2*

NM_010708.2	*Lgals9*
NM_007648.5	*Cd3e*
NM_001081110.2	*Cd8a*
NM_010136.3	*Eomes*
NM_008399.3	*Itgae*
NM_001167906.2	*Cd101*
NM_030710.3	*Slamf6*
NM_009327.3	*Hnf1a*
NM_009987.4	*Cx3cr1*
NM_009910.3	*cxcr3*
NM_008390.2	*Irf1*
NM_001205313.1	*Stat1*
NM_013488.3	*Cd4*
NM_019507.2	*Tbx21*
NM_011487.5	*Stat4*
NM_008354.4	*IL12rb2*
NM_008091.3	*Gata3*
NM_011488.3	*Stat5*
NM_001008700.4	*Il4r*
NM_013674.2	*Irf4*
NM_011281.3	*Rorc*
NM_213659.3	*Stat3*
NM_001199347.1	*Foxp3*
NM_013693.3	*Tnfa*
NM_010735.2	*Tnfb*
NM_013542.3	*Gzmb*
NM_010370.3	*Gzma*
NM_011073.3	*Prf1*
NM_008366.3	*Il2*
NM_008337.4	*Ifng*
NM_021283.2	*Il4*
NM_031168.2	*Il6*
NM_008361.4	*Il1b*
NM_010552.3	*Il17a*
NM_021782.3	*Il21*
NM_010548.2	*Il10*
NM_010554.4	*Il1a*
NM_011045.2	*Pcna*
NM_001379248.1	*Ccnd1*
NM_001081117.2	*Mki67*
NM_008753.4	*Oaz1*
NM_013854.1	*Abcf1*
NM_011655.4	*Tubb5*
NM_008062.2	*G6pdx*
NM_023281.1	*Sdha*

NM_001291068.1	*Polr2a*
NM_020559.2	*Alas1*
NM_013684.3	*Tbp*
NM_183155.2	*Nrde2*
NM_010368.1	*Gusb*
