# Supplementary figures and images for: Co-targeting TGF-β and PD-L1 sensitizes triple-negative breast cancer to experimental immunogenic cisplatin-eribulin chemotherapy doublet
Source: J Clin Invest. 2025 Jul 1;135(13):e184422. doi: 10.1172/JCI184422 (PMC12208543; doi:10.1172/JCI184422)

Figure 2

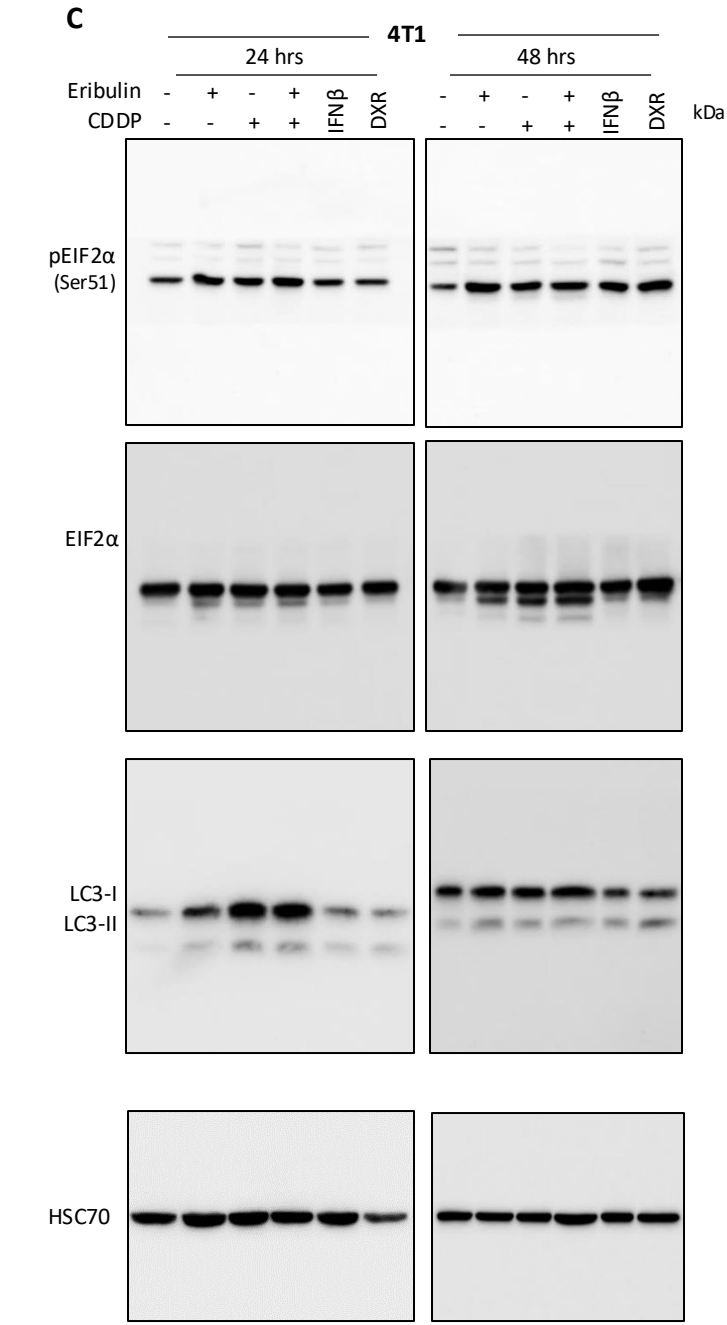

Figure 4

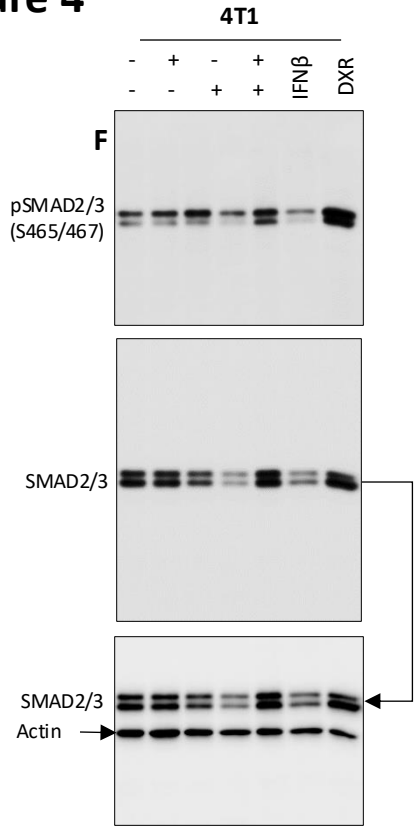

Supplement: Unedited blot and gel images [file jci-135-184422-s231.pdf]
